# Transdermal needle-free drug delivery approaches and activation mechanism

**DOI:** 10.1016/j.mtbio.2026.103002

**Published:** 2026-03-05

**Authors:** Apoorva Sasikala, Du Tuan Tran, Jun Zhang, Nam-Trung Nguyen

**Affiliations:** a Queensland Quantum and Advanced Technologies Research Institute, Griffith University, Nathan, QLD, 4111, Australia; b School of Engineering and Built Environment (EBE), Griffith University, Nathan, QLD, 4111, Australia

**Keywords:** Transdermal drug delivery, Needle-free injection, Permeation enhancers, Activation mechanisms, Skin, Cavitation

## Abstract

The evolution of transdermal drug delivery (TDD) systems has transformed non-invasive drug delivery by improving safety, precision, and patient compliance. While conventional TDD methods face challenges with penetration efficiency and dosage control, needle-free transdermal drug delivery technologies are emerging as a promising alternative. This review critically examines recent advancements in transdermal needle-free drug delivery technologies, focusing on different physical penetration methods and activation mechanisms such as spring-based, gas-driven, electrochemical, piezoelectric, laser-induced and electromagnetic approaches, which generate sufficient force for increasing the penetration ability of the drugs through the stratum corneum. The paper also discusses the implications of these technologies for wearable and personalised healthcare, highlighting future directions for clinical translation and real-world deployment.

## Graphical abstract

**Figure:**
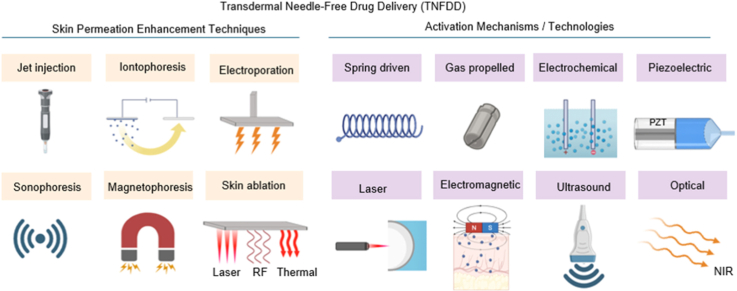

## 1 Introduction

Advancing drug delivery systems is a fundamental pillar of modern medicine, as improving bioavailability, targeting precision, and controlled release leads to fewer side effects and better patient experience. Drug delivery systems are fundamentally designed to transport active pharmaceutical ingredients to targeted sites in the human body while controlling the timing, rate, and duration of their release [1]. The clinical practice continues to rely on conventional administration routes, despite the significant progress in drug delivery technologies. The two main administration routes are oral and parenteral delivery. Both routes face built-in restrictions, which negatively affect both therapeutic results and operational performance. Oral delivery offers convenience in drug intake, but its bioavailability is limited due to enzymatic degradation in the gastrointestinal tract and extensive first-pass hepatic metabolism [2,3]. Parenteral delivery methods, including intravenous (IV) and intramuscular injections, provide better pharmacokinetic predictability and superior bioavailability. However, the invasive nature of parenteral delivery methods leads to pain and infection risks as well as increased procedural expenses due to the requirement of sterile conditions and trained healthcare staff during the delivery process. In addition, the improper use of needles might spread bloodborne infections like HIV and HBV, thus demonstrating the immediate need for safer delivery methods [4,5]. In response to these challenges, non-conventional pathways like inhalation, vaginal or intraosseous administration have been explored, but these techniques also have their own drawbacks, such as discomfort, mucosal distress, or extensive maintenance requirements [[6], [7], [8]].

The COVID-19 pandemic has motivated a trend towards decentralised and home-based care, in favour of self-administration and telemedicine [9]. A 2021 global survey indicated that 61% of Chinese and 33% of US clinicians predicted that most healthcare services would be provided in people's homes by 2031 [10]. This paradigm shift calls for the development of easy-to-use and efficient drug delivery devices that will enable patients to administer their therapies outside conventional clinical environments. In answer to these developments, transdermal needle-free drug delivery systems (TNFDDS) have emerged as a transformative solution. These technologies eliminate the use of needles, and thus avoid needle-related pain, anxiety, distress and risk of infection [11]. Moreover, their capability of being self-administered enhances the degree of patient control and compliance, while at the same time lowering the costs of healthcare and demand on clinical personnel resources. TNFDDS are particularly well-suited for mass immunisation and decentralised care with benefits spanning from advanced hospital settings to resource-poor environments with limited staff and infrastructure.

**Fig. 1:**
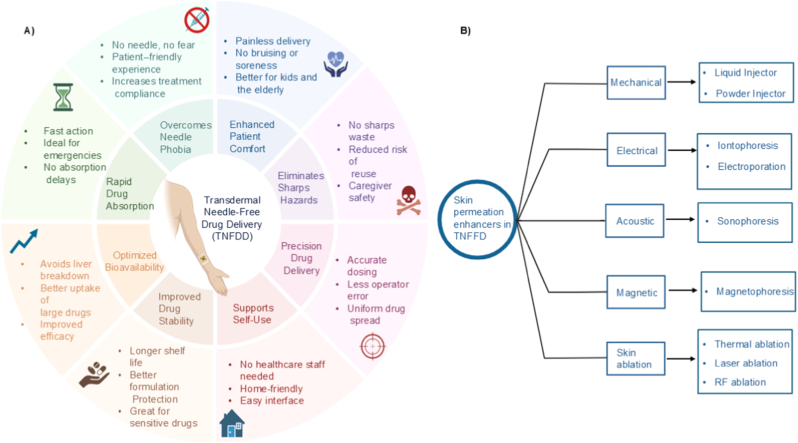
Overview of transdermal needle-free drug delivery systems: A) Advantages of needle-free transdermal drug delivery; B) Classification of skin permeation enhancement mechanisms in transdermal needle-free drug delivery.

Fig. 1A depicts the main advantages of TNFDDS. A range of techniques have been developed to facilitate needle-free transdermal delivery, and these include jet injection systems, transdermal patches and energy-based drug delivery techniques such as iontophoresis, sonophoresis (ultrasound-enhanced permeation), electroporation, and cutaneous ablations (Fig. 1B) [12]. These platforms stand out from the conventional techniques due to their high-precision activation mechanisms, which provide the energy required to overcome the stratum corneum [13]. These actuators convert stored energy from springs, gases, electromagnetic or thermal sources into mechanical force and effectively deliver drugs through the skin [14]. The actuator design and the performance can directly influence the crucial delivery parameters such as jet velocity, penetration depth, dose uniformity, and delivery efficiency [15].

Despite the above promising developments, integrating transdermal needle-free drug delivery systems (TNFDDS) into compact, wearable, and intelligent formats remains challenging. Achieving this requires an interdisciplinary effort spanning microfluidics, materials science, and device engineering [16,17]. While numerous reviews emphasise clinical use and therapeutic outcomes, fewer address the biophysical activation principles that ultimately define delivery performance, controllability, and safety. In particular, understanding how devices generate and regulate propulsion or energy inputs, through gas pressure, spring loading, electromagnetic actuation, or piezoelectric pulses, provides direct insight into scaling potential and platform limitations.

In several prior reviews, microneedles are described as “needle-free” and minimally invasive; however, they still breach the skin barrier. Accordingly, this review focuses on needle-free transdermal delivery without microneedles and excludes microneedle-assisted and hybrid microneedle systems. Within this scope, three technology families are considered such as propulsion-based delivery that traverses the barrier (liquid jet and powder/biolistic injection), physical energy–based permeability enhancement (electrical, acoustic, magnetic, thermal, and optical), and activation technologies that generate and regulate these energy inputs (spring-driven, gas-powered/combustion-based, electrochemical, electromagnetic/Lorentz-force, piezoelectric, and optically/laser-triggered systems). The literature prioritised here is peer-reviewed and reports quantitative delivery performance (e.g., penetration depth, flux, delivered dose) and/or quantitative activation outputs (e.g., pressure/force profiles, jet or particle velocity, pulse waveform parameters, acoustic intensity, thermal dose), together with barrier disruption and recovery markers, safety outcomes, and translational evidence. Studies centred on conventional hypodermic needles or primarily chemical enhancement were not considered, and non-transdermal or implantable routes were included only when they provided activation outputs directly transferable to TNFDDS device design and control. By connecting these operating-condition metrics to formulation-relevant constraints, this review provides a practical basis for formulation scientists to define modality-appropriate critical quality attributes (CQA)s and to de-risk translation through device–formulation matching, including choices in excipients, concentration and viscosity windows, solid-state stability, and packaging strategy.

Accordingly, this review critically examines the TNFDDS landscape, with emphasis on physical enhancement strategies, activation mechanisms, and emerging clinical applications. To enable cross-comparison and improve readability, the review first introduces core engineering principles, then summarises physical enhancement techniques, and finally analyses activation mechanisms. The practical implementation of these strategies is discussed to highlight translational potential.

## 2 Skin physiology and its impact on needle-free transdermal delivery

**Fig. 2:**
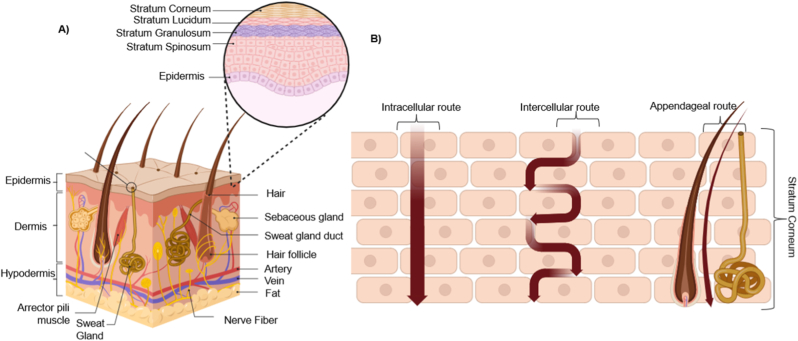
Structure of the skin and pathways of transdermal drug transport. A) Schematic representation of skin structure showing the epidermis, dermis, and hypodermis with associated appendages; B) illustration of drug permeation pathways across the stratum corneum.

The skin, the largest organ of the human body, serves as both a regulatory and protective interface. Its multi-layered structure, consisting of an outer epidermal and underlying dermal and hypodermic layers, influences needle-free transdermal drug delivery [18]. As shown in Fig. 2A, the outermost epidermal layer is secured by the stratum corneum (SC), the skin's principal barrier to drug absorption. The SC is composed of flattened, dead keratinocytes embedded in a lipid matrix, serves as a 'brick-and-mortar' structure. The stratum corneum is typically 10 to 30 μm thick, and its hydrophobic composition limits the penetration of hydrophilic and large-molecule compounds [19]. Passive transdermal diffusion of drugs happens through one of three pathways: transcellular, intercellular, or appendageal (Fig. 2B) [20]. Hydrophilic drugs prefer the transcellular route, while lipophilic compounds prefer the intercellular route [21]. However, this process is limited by the SC's selective permeability, which poses a particular challenge for hydrophilic molecules. Additionally, appendageal routes, such as through hair follicles and sweat glands, provide alternate portals that may bypass the SC's densely packed barrier, especially for larger or polar substances [22].

Beneath the epidermis lies dermis, a densely packed layer with a network of connective tissue, capillary vessels. The dermis also averages in thickness 1 to 4 mm [23]. The drug molecules, after passing through SC, can reach and diffuse into the dermis, entering systemic circulation by capillary uptake [24]. Underneath the dermis is the hypodermis, consisting primarily of fat and connective tissue, which also plays a role in thermoregulation as well as cushioning [25]. Though the hypodermis is not a major site of drug absorption, it can act as a reservoir for controlled release of lipophilic drugs. Various biological factors of the skin influence the efficacy of transdermal drug delivery systems [18]. Increased hydration of the skin improves permeability through disruption of lipid packaging. Aging alters skin diffusion potential and morphology, while inflammatory disorders or lesions have the potential to enhance or impair drug flux. The location of drug application also matters, since the skin permeability varies across the body [26,27].

## 3 Skin permeation enhancement techniques

Passive diffusion has long served as the conventional route for transdermal drug delivery. However, the process is inherently slow and limited in efficiency due to the strong barrier properties of the SC, which significantly restrict the amount and rate of drug transport into deeper skin layers. Overcoming this barrier is therefore critical for achieving effective and reproducible drug delivery, making skin permeation enhancement techniques central to transdermal therapies. In this session, we focus exclusively on physical enhancement strategies, deliberately excluding chemical enhancers due to their limited controllability, variable efficacy, and potential for skin irritation [28]. While many physical methods aim to enhance skin permeability transiently, certain needle-free platforms, such as jet and powder injection systems, deliver drugs directly through the skin barrier. These approaches are included here due to their relevance as needle-free transdermal delivery, even though they operate via direct drug propulsion rather than modifying the skin barrier itself.

TNFDD systems employ precisely applied physical forces, ranging from mechanical propulsion to electromagnetic or acoustic energy, to create temporary pathways for therapeutic agents to enter viable skin layers. These platforms are designed to deliver reproducible doses within seconds, with penetration depth and dispersion pattern determined by finely tuned triggering parameters [29]. The TNFDDS encompasses a range of modalities, each with distinct working principles and engineering limitations [30]. According to their working principles and the type of energy applied, skin permeation enhancement techniques can be broadly classified as mechanical, electrical, acoustic, magnetic, thermal, and optical methods.

**Fig. 3:**
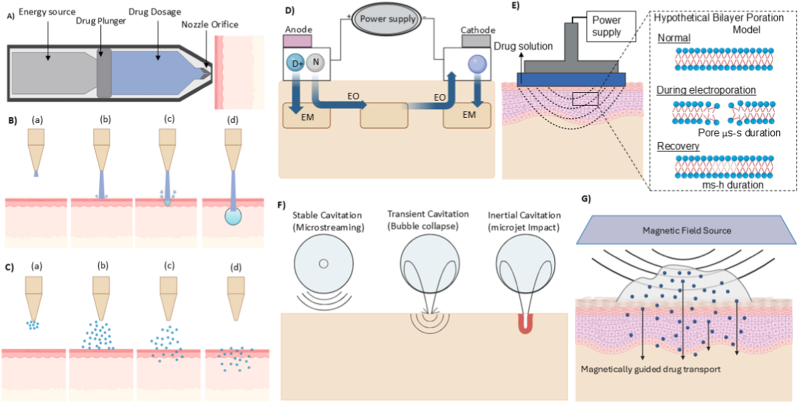
Overview of various skin permeation enhancement techniques: A) Schematic of jet injector device delivering drugs through the skin; B) Jet injection mechanism stages: (a) approach, (b) initial contact, (c) penetration, and (d) drug deposition; C) Powder injection process with particle dispersion stages; D) Iontophoresis using electrical current to facilitate drug delivery across the skin via electroosmosis (EO) and electromigration (EM); E) Electroporation setup and bilayer poration model showing transient pore formation to enhance drug permeability; F) Mechanisms of ultrasound-mediated delivery: stable cavitation, transient cavitation, and inertial cavitation; G) Magnetically guided drug delivery using external magnetic fields to direct drug particles across the skin barrier.

The mechanical approach relies on kinetic energy to puncture through the skin barrier. **Jet injection** is a rapidly evolving mechanical method for needle-free transdermal drug delivery [15]. The drug is delivered in suspension form through a high-velocity jet ejected from a thin nozzle. Intradermal, subcutaneous, or intramuscular injections can be administered by adjusting injection parameters such as jet velocity, fluid pressure, nozzle size, standoff distance, and injection angle [31,32]. Jet injection can be classified into liquid jet injectors and powder jet injectors, according to the physical state of the drug. **Liquid jet injectors** comprise three integrated components: a drug reservoir, a nozzle, and a power source [33]. Fig. 3A illustrates the core components and operational mechanism of a needle-free liquid jet injector. The liquid drug formulation is held in a reservoir connected to a nozzle. The nozzle diameter ranges from 76 to 360 μm [33]. The power source provides the energy required to accelerate the liquid-based drug [34]. Upon activation, this energy is transferred via a diaphragm or a piston, which rapidly increases the pressure within the drug chamber, creating a jet of liquid with a speed of approximately 100 m/s [16]. To achieve effective skin penetration, liquid jet injection relies on turbulent flow, where the Reynolds number (Re) often reaches the order of tens of thousands, generating a highly focused, high-energy jet capable of breaching the skin barrier [35].

Jet penetration occurs in two distinct phases: an initial high-pressure spike forms a microchannel in the skin, followed by lateral diffusion of the drug as the jet speed decreases [34]. The resulting delivery is predominantly spherical in profile. Due to the rapid timescale, in the order of microseconds, high-speed imaging or ultrasound is required to visualise this process. Fig. 3B provides a visual representation of jet formation and subsequent injection into the skin tissue [36,37]. Optimising exit power is essential for maximising drug deposition within the tissue, as it directly influences the efficiency and completeness of delivery. Exit power, defined as:

(1) $$\begin{array}{c} P=\frac{1}{8}\pi \rho D^{3}V^{3} \end{array}$$

increases exponentially with jet velocity (*V*) and nozzle diameter (*D*), making it a critical design variable [36]. Additionally, tissue stiffness, quantified by Young's modulus, plays a major role: stiffer skin resists penetration and reduces delivery completeness, whereas softer tissue facilitates deeper and more efficient deposition [38].

**Powder injections** are conceptually related to liquid jet injections, but they operate by accelerating dry, solid microparticles at high velocity to mechanically breach the skin barrier and deliver the drug into the epidermis or dermis [33,39]. The term “ballistic” or “biolistic” delivery is often used in reference to needle-free powder injector (NFPI) systems because they rely on high-velocity particle propulsion rather than fluid dynamics [40]. Unlike liquid jet systems, which depend on hydrodynamic forces, powder injections depend entirely on the kinetic energy of solid particles to overcome the skin barrier. Similar to the jet injectors, a powder injector has three essential components: an actuator, a drug reservoir, and a nozzle. As the particles exit the nozzle, they are accelerated to velocities exceeding 200 m/s, allowing them to perforate the SC and generate transient microchannels in the epidermis. Successive particles follow the same trajectory and accumulate at the base of the microchannels, forming a subepidermal drug depot (Fig. 3C) [37,41]. The drug is then slowly released and absorbed, depending on parameters such as solubility, particle size (10 to 20 μm in diameter), and local skin hydration [42,43]. For effective drug delivery, smaller particles are often coated with high-density metals like gold or tungsten to increase their momentum, which enhances skin penetration and ensures more accurate delivery [44]. For example, gold microparticles have achieved penetration depths ranging from 35 to 135 μm under a driver pressure of 3 MPa [45]. Effective particle densities for successful delivery typically range from 1.08 to 18.2 g/cm^3^ [39]. The diameter of the treated area also affects the performance, with most NFPI systems being optimised for skin regions approximately 4 mm in diameter. A useful engineering metric to evaluate NFPI performance is the impact parameter (ρ*vr*), which is defined as the product of particle density (ρ), velocity (*v*), and radius (*r*). This parameter represents the particle's momentum per unit cross-sectional area and correlates strongly with its ability to penetrate the outer skin barrier. Higher impact parameter values generally lead to deeper penetration and improved payload deposition within viable tissue [46,47].

While mechanical approaches rely primarily on kinetic energy to overcome the SC barrier, electrical techniques offer a controllable and reversible means to modulate skin permeability through electrokinetic effects. **Iontophoresis** employs a mild electric current to facilitate the movement of ionic and polar therapeutic agents across the SC. This technique relies on the electrostatic repulsion and electrokinetic phenomena to transport the hydrophilic and charged molecules [48]. When the electric field is applied across the skin, the dipole molecules (proteins and lipids) in the skin will rotate and align with the field, causing temporary nano pores. Thus, the drug will be induced into the skin through these pores rather than the natural pores of the skin [49]. Iontophoresis is controlled by several factors, such as intensity and duration of the applied current and the type of input [50]. The concept enhances molecular transport across biological barriers primarily through electromigration, electroosmosis, and, to a lesser extent, passive diffusion [51]. A typical iontophoretic system consists of a microprocessor-controlled power unit and two electrodes: an active electrode containing the drug formulation and a return electrode completing the circuit [52,53]. Drug placement depends on charge polarity, with positively charged drugs under the anode, negatively charged under the cathode, allowing for precise local penetration [41]. Fig. 3D illustrates schematically this setup. Electrodes made from silver–silver chloride (Ag/AgCl) enhance stability and biocompatibility; redox reactions at the electrodes maintain charge balance and minimise skin irritation [54]. By dynamically modulating the applied current, iontophoretic systems enable real-time control over transdermal flux, allowing precise adjustment of dose rate and total delivered amount to match individual patient requirements. For instance, pharmacokinetic studies report drug appearance in the bloodstream within approximately 2.5 min, which is significantly faster than that achieved through oral administration [55]. Operational factors strongly influence iontophoretic efficacy. Among these, the transport number (*t*_D_) quantifies the fraction of the applied current that drives drug molecules. A *t*_D_ value of 1 indicates complete utilisation of the current for drug transport, whereas values near zero suggest that other ions dominate conduction, thereby limiting drug movement [56]. This complexity means that increasing the current density does not always yield a proportionate increase in flux. Transport efficiency is influenced by drug concentration, ionic mobility, valence, and membrane interactions, which can sometimes result in plateau effects [57].

**Electroporation** is another electrical enhancement method used for the temporary disruption of the SC by employing short high-voltage electric pulses. Aqueous micropores will be formed in the lipid matrix of the SC, which enhances the permeation of hydrophilic and high-molecular-weight molecules [58]. Even though electroporation and iontophoresis both employ electrical energy to enhance skin permeability, their mechanisms and effective drug options are quite different. In practice, electroporation relies on delicate optimisation of device shape and pulse parameters to optimally balance pore generation while inhibiting irreparable tissue damage [59]. The most significant parameter of the effectiveness of electroporation is the waveform of the pulse at delivery. Square-wave pulses are popular due to their ability to produce uniform and prolonged electric fields, which promote consistent and predictable pore generation throughout the treatment site [60]. In contrast, exponentially decaying pulses, often produced by discharge from a capacitance, deliver an initially high-voltage peak and subsequently taper off, enabling long periods of lower-intensity electric field treatment. This long 'tail' provides benefits in maintaining prolonged molecular transport once initial permeabilisation has taken place [61]. In either case, the amplitude of the applied voltage must be greater than a certain threshold (30-100 V) to effectively disrupt the intercellular lipid matrix. Below this threshold, pore formation is insufficient to facilitate significant drug flux; on the other hand, excessively high fields may trigger localised heating, irreversible lipid phase transitions, or cytotoxicity [62]. The transport of molecules across the electroporated skin proceeds through multiple mechanisms [58,63].

Fig. 3E depicts a schematic illustration of a typical electroporation with pore formation. Pulse duration, frequency, and interval spacing are other critical parameters in modulating electroporation. Parameter selection depends on the drug formulation and is influenced by size, charge, and physicochemical properties. Small hydrophilic molecules may require shorter pulses to prevent excessive disruption, while macromolecular biologics involve multiple pulses to overcome their inherent permeability barriers. Electrode design is another determining factor in optimising the system. The spatial arrangement of electrodes regulates the local field electric distribution, which in turn determines the uniformity and depth of skin permeabilisation [64]. Non-invasive geometries, such as flat plaques or flexible patches, are often employed in wearable devices due to their simplicity in construction and low tissue reactivity [65]. Material selection for electrode and reservoir components is critical to device performance, as it affects electrochemical stability and conductivity. Charge density also plays a key role, since highly charged molecules respond more strongly to electrophoretic forces, with their mobility governed by the molecule's acid dissociation constant pKa and the medium's pH. This makes pH a critical parameter not only for controlling the stability and bioactivity of drugs, but also for modulating the responsiveness of the skin barrier [66]. In addition, co-administration of ionic excipients, such as monovalent (e.g., NaCl, NaI) or divalent (e.g., CaCl_2_, MgCl_2_) salts, has also been shown to deliver superior electroporation effects [67].

Acoustic energy provides yet another non-invasive alternative. **Sonophoresis**, also referred to as phonophoresis, enhances skin permeability by applying controlled ultrasound waves, which transiently disrupt the SC and facilitate the transport of drug molecules across it. This approach relies on high-frequency acoustic energy ranging from 20 kHz to 16 MHz [41,68]. One of the key advantages of sonophoresis over other physical enhancement techniques, such as iontophoresis or electroporation, lies in its independence from drug charge. In contrast to methods that rely on electrical currents and are thus limited to ionisable compounds, sonophoresis can effectively transport both neutral and charged molecules, encompassing both hydrophilic and lipophilic drug classes [69,70].

Sonophoresis can be employed using two approaches: simultaneous application and pretreatment. In the simultaneous approach, ultrasound and drug molecules are applied at the same time; the acoustic waves continuously perturb the skin, allowing the drug to diffuse more easily [71]. In contrast, the pretreatment approach involves applying ultrasound to the target site first to temporarily disrupt the stratum corneum, followed by drug administration. At higher intensities or frequencies, ultrasound leads to localised heating, which raises skin permeability due to enhanced lipid fluidity and molecular diffusion. However, thermal contributions are relatively modest compared with the dominant mechanical effects of ultrasound [41]. A 35-fold enhancement in mannitol transport using 20 kHz ultrasound has been reported, primarily attributed to cavitation, the dynamic behaviour of microscopic gas bubbles within the skin's aqueous microdomains (Fig. 3F) [72,73].

Cavitation occurs in two principal forms: stable cavitation, where oscillating bubbles induce microstreaming and shear forces that disrupt lipid packing, and inertial cavitation, where violent bubble growth and collapse generate shock waves, microjets, and transient local increases in temperature and pressure [74]. These intense mechanical events create nanoscale aqueous channels in the SC and can even dislodge lipids, thereby providing transient but effective conduits for drug transport. Cavitation probability greatly depends on acoustic parameters, which are summarised as the Mechanical Index (MI): peak negative pressures increase cavitation potential, and higher frequencies suppress it [75]. In addition to cavitation, ultrasound forms localised transport regions (LTRs), micron-sized SC areas with very high permeability [76]. These heterogeneous “hot spots” act as preferential gateways for molecules, amplifying the overall delivery efficiency. Furthermore, coupling media such as hydrogels or aqueous solutions not only transmit acoustic energy effectively but also provide a reservoir of drug that can be actively propelled into the skin. From an engineering standpoint, sonophoresis devices consist of piezoelectric transducers, usually of quartz or lead zirconate titanate (PZT), converting electrical pulses into mechanical oscillations of varying frequency and amplitude [77]. Wearable sonophoresis devices typically integrate a transducer, power/control unit (battery and microcontroller), hydrogel or drug reservoir as the coupling medium, and a biocompatible skin-contacting material for effective acoustic transmission [78]. Thin-film transducers operating at 20–100 kHz provide flexibility and effective cavitation, enabling seamless wearable integration.

Beyond acoustic stimulation, magnetic fields have also been investigated as non-contact driving forces for drug delivery. **Magnetophoresis** is an emerging needle-free skin permeation technology that employs magnetic fields to facilitate the transport of therapeutic agents [79]. The core mechanism underlying magnetophoresis is diamagnetic repulsion. Diamagnetic substances, characterised by their lack of unpaired electrons and negative magnetic susceptibility, are inherently repelled by magnetic field [80]. Fig. 3G illustrates the schematically the magnetophoretic flow of drug molecules away from the magnetic source and into the SC. This fundamental property enables the application of external magnetic fields to non-invasively drive drugs through biological barriers [81]. Magnetophoresis can be broadly classified into two modalities: static and pulsed. Static magnetophoresis employs continuous magnetic fields generated by permanent magnets, while pulsed electromagnetic fields (PEMFs) leverage alternating currents to induce time-varying magnetic fields [82]. According to Faraday's law, these fields can generate weak electric currents within tissues, transiently increasing membrane permeability by forming ionic channels. PEMFs have also demonstrated utility in tissue regeneration, including bone healing and the ability to change the permeability of the SC. Benson et al. reported a study involving the deposition of gold nanoparticles (10 nm) on both PEMFs-treated and non-treated skin [81]. The PEMF-treated skin absorbed 200 times more gold nanoparticles than the untreated skin. This suggests that the electromagnetic pulses significantly increase the skin's permeability.

Magnetophoretic drug delivery devices typically comprise a drug reservoir, a magnetic field source, and a skin-contact interface. Static systems utilise permanent magnets, whereas pulsed systems employ electromagnetic coils to generate time-varying fields. For safe application, static magnetic fields for transdermal delivery are generally kept within 100–500 mT, which effectively enhances drug permeation without causing structural or thermal damage to the skin. Drug delivery via magnetophoretic devices generally follows two strategic pathways. The first involves attaching drug molecules to magnetic carriers, typically iron oxide nanoparticles, and guiding them to target tissues using externally applied magnetics gradients [83]. This strategy was demonstrated in a study where Fe_3_O_4_ nanoparticles were synthesised and coated with polyvinyl alcohol, layered double hydroxide (Zn/Al-LDH), and the anticancer drug sorafenib. The coated nanoparticles retained their magnetic responsiveness and exhibited enhanced cytotoxicity against HepG2 liver cancer cells, while remaining non-toxic to normal fibroblast cells [84]. The second approach relies on direct magnetic repulsion to improve transdermal drug diffusion, where static magnetic fields in the range of 1 to 5 × 10^2^ mT are applied, safe for skin application [85].

Thermal, optical (laser), and radiofrequency (RF) methods are employed to enhance skin permeation. All three rely on the same principle: controlled, partial, and reversible disruption of the SC to create transient microchannels that facilitate drug transport. **Thermal ablation** enhances transdermal drug delivery by selectively and precisely disrupting the skin's outermost barrier through brief, localised heating. This targeted approach temporarily increases skin permeability [86]. Rather than bypassing the barrier entirely, thermal ablation removes microdomains of the SC in a controlled manner, creating transient microchannels that facilitate molecular diffusion. **Laser ablation** is another ablation technique, which employs precisely directed laser energy to enhance transdermal drug delivery. Like thermal ablation, laser ablation relies on the rapid evaporation of intercellular water molecules within SC, resulting in the formation of micropores to facilitate molecular transport [87]. The ablation performance and tissue specificity are critically dictated by the laser's operating wavelength. Although wavelengths near 2490 nm are commonly employed, maximal water absorption occurs at 2790 nm, and protein-selective ablation is optimally achieved at 2940 nm [88]. The mechanism relies on flash evaporation, the water and proteins in the skin will rapidly absorb the laser energy, creating localised micro explosions that in turn ablate the tissue and form micropores with a 150-200-μm diameter [89]. The depth and extent of tissue ablation can be finely controlled by adjusting laser parameters, including pulse energy, duration, and repetition rate [90]. Lower energy exposures lead to partial SC removal, while higher fluences permit deeper penetration into the viable epidermis or upper dermis. Two laser classes have demonstrated optimal performance: short-wavelength ultraviolet lasers, which target protein absorption, and mid-infrared lasers, which interact primarily with water [91,92]. Due to the localised and ultrafast nature of laser delivery, surrounding tissues are spared from thermal damage. The resulting micropores form transient aqueous channels that enable enhanced transdermal flux of hydrophilic and macromolecular drugs. Notably, the confined energy deposition minimises collateral tissue heating, preserving skin integrity and ensuring safety. Despite its demonstrated advantages in permeability enhancement and precision, laser ablation remains underrepresented in literature, with limited studies evaluating its translational potential in drug delivery applications.

**Radiofrequency (RF) ablation** is another way that enhances transdermal drug penetration through controlled thermal lysis by applying high-frequency AC currents. Typically, AC in the range from 100 kHz to hundreds of MHz is applied, inducing quick ionic motion in tissues [93]. Unlike other electrical permeation techniques, RF ablation causes frictional heating and subsequent localised thermal damage, leading to evaporation of intracellular water and the formation of microchannels within the SC. Short pulses of energy are delivered in RF ablation to prevent excessive heating. Modern systems often incorporate sensors to monitor and adjust power output in real time [94]. RF systems are generally categorised as monopolar, bipolar, and fractional RF designs. Monopolar systems direct current from the treatment electrode into the body to a distant body site for grounding, thus creating energy to penetrate to deeper tissues [95]. In contrast, bipolar systems have electrode pairs on the skin surface in very close proximity to one another, restricting current flow to the region between them and consequently restricting heat production to shallower tissue levels. This localised and controlled energy deposition makes bipolar RF particularly suitable for transdermal drug delivery [96]. Fractional RF, a further refinement, employs an array of microelectrodes to deliver energy in a pixelated pattern. This approach produces discrete zones of thermal ablation interspersed with undamaged skin, thereby facilitating rapid healing while maintaining permeability [97].

The physical enhancement and propulsion-based strategies discussed in this section can be reframed as different ways of delivering energy to the skin–drug interface to overcome the SC barrier. Mechanical momentum (jet and powder), electric fields (iontophoresis and electroporation), acoustic cavitation (sonophoresis), magnetic driving forces (magnetophoresis), and localized heating/ablation (thermal, laser, and RF) all converge on a common set of outcomes that define penetration depth and deposition pattern, the duration of barrier disruption and recovery kinetics, and the safety window indicated by histological or functional endpoints. Importantly, these outcomes are not determined by the permeation mechanism alone, but they depend on how precisely the required energy is generated, triggered, and regulated within the device.To support cross-comparison, Table 1 summarises the key controlling parameters, depth of interaction, disruption/recovery kinetics, payload suitability, and the evidence basis reported across these modalities. Therefore, the next section shifts from barrier-level mechanisms to the engineering mechanisms that power them, by classifying and analysing activation technologies that enable controlled, reproducible needle-free drug delivery.

**Table 1:** Classification framework for needle-free technologies based on penetration depth, barrier disruption duration, recovery time, and strength of supporting evidence.

Sl No.	Technology	Controlling parameters	Penetration depth	Barrier disruption duration	Recovery time	Type of drug administered	Histological evidence	Ref
1	Liquid Jet Injector	⁃ Nozzle diameter: ∼100–200 μm ⁃ Jet velocity: >100 m/s ⁃ Flow rate: ∼1 mL/s	⁃ Intradermal/dermal deposition: 2 ∼4 mm ⁃ Deeper deposition IM targeting ∼3 cm	⁃ Immediate formation of a microcavity/track along the jet path ⁃ The persistence of barrier impairment is not consistently quantified across studies.	⁃ Short-lived local reactions in clinical use. ⁃ Quantitative barrier recovery kinetics are inconsistently reported	⁃ Vaccines ⁃ Insulin ⁃ local anaesthetics ⁃ dermal injectables such as hyaluronic acid-based products	⁃ Histology/fluorescence imaging commonly shows a jet-formed cavity/track with dermal dispersion ⁃ Supports classifying jet injection as minimally invasive (micro-track formation) rather than fully non-invasive.	[[98], [99], [100]]
2.	Powder Jet Injection	⁃ Particle diameter 0.5–3 μm (DNA vaccination), 10–20 μm (most drug powders) ⁃ Particle density - 1.08–18.2 g/cm^3^ ⁃ Impact velocity ∼600–900 m/s	⁃ 35 to 95 μm for 1.8 μm particles ⁃ 5 to 135 μm for 5 μm	⁃ Instantaneous barrier breach (impact on the order of 100 μs)	⁃ Typical use- generally reversible within ≤4 days, and no later than 10–14 days (worst-case window reported ⁃ Higher-intensity dosing: effects reversed within ≤24 h. ⁃ Irritation scores often return to baseline rapidly (e.g., by day 1 in some reports)	⁃ DNA/pDNA vaccines on microcarriers ⁃ Protein antigens ⁃ Small-molecule powder example: lidocaine	⁃ Superficial epidermal disruption (keratin debris/crust; mild injury markers) with limited dermal involvement. ⁃ Particles detected in viable epidermis ⁃ Some studies reported intracellular localisation (e.g., gold by TEM)	[101,102]
3.	Iontophoresis	⁃ Current density (J = 0.1-0.3 mA/ ${cm}^{2}$ ⁃ Duration = 5-15 min ⁃ Electrode configuration	⁃ No physical microchannel ⁃ primary barrier interaction at the SC, ∼10–20 μm with enhanced transport across SC pathways	⁃ During current delivery – 5-15 min ⁃ Post effect window depends on dose i 2h at 0.5 mA/ ${cm}^{2}$ ii 48h at 0.13mA/ ${cm}^{2}$	⁃ ∼30 min – impedance recovery trend after 15 min exposure ⁃ ∼ 1h – microvascular and blood flow changes return to normal after 30 min of exposure with an intensity of 0.1 – 0.2 mA/ ${cm}^{2}$ ⁃ ≥ 18h – Impedance recovery after 2h exposure at 0.5mA/ ${cm}^{2}$	⁃ Ions/polar drugs (cations/anions) via electromigration ⁃ Neutral/weakly charged molecules via electroosmosis	⁃ Electron microscopy and histology report SC pathway changes (e.g., intercellular dilation) ⁃ Disturbance of epidermal calcium gradients ⁃ Disruption is current-density dependent and is reported as reversible at lower intensities	[[103], [104], [105], [106], [107], [108]]
4.	Sonophoresis	⁃ Frequency: i LFS 20–100 kHz ii HFS: 0.7–16 MHz ⁃ Acoustic intensity: 0.1–3 W/cm^2^ ⁃ Coupling medium: water/gel ⁃ duty cycle: continuous or pulsed	⁃ Primary barrier affected: SC, ∼15 μm ⁃ Beyond SC evidence: 20 nm quantum dots detected beyond the SC after LFS	⁃ During exposure: minutes ⁃ Post-effect window: enhanced permeability can persist up to 24h ⁃ Human in vivo: Permeability remained high for ∼42h under occlusion	⁃ Water permeability: 94% of the change recovered within 2h after ultrasound termination ⁃ Barrier recovery after de-occlusion: rapid onset; impedance increases immediately after occlusion removal	⁃ Macromolecules delivered: insulin ⁃ Low- molecular weight heparin ⁃ Vaccines ⁃ Clinical drugs reported: Lidocaine, Cyclosporin	⁃ Clinical observation (human): no erythema, no edema, and no adverse events during the monitored period ⁃ Safe window (human; 20 khz): ≤2.5 W/cm^2^ reported as macroscopically normal with no structural disruption ⁃ Damage thresholds: i 4 W/cm^2^, continuous, 10 min: epidermal separation and dermal necrosis reported ii 7 W/cm^2^ continuous and 12.3 W/cm^2^ pulsed: second-degree burns reported	[[109], [110], [111], [112], [113]]
5.	Electroporation	⁃ Pulse voltage: 50-3000V (common range: 5-500V) ⁃ Pulse duration: 5μs- 100 ms ⁃ Pulse number: 10-100 ⁃ Frequency: 1-5Hz ⁃ Electrode spacing ⁃ Conductive gel/hydrogel electrode	⁃ Primary barrier affected: stratum corneum (SC) 10–15 μm. ⁃ delivery facilitated into viable epidermis 50–100 μm ⁃ SC disruption reported at ∼100 V	⁃ During pulse train (ms–s total). ⁃ Post-effect window: pores persist ≥2 h ⁃ enhanced permeability reported >12 h (occlusion can extend the effect).	⁃ Mild non-invasive protocols: TEWL restoration ∼5 min reported. ⁃ Higher-energy protocols: recovery can extend to hours (≥2 h to >12 h, protocol dependent).	⁃ Small hydrophilic drug ⁃ Ions and polar drugs ⁃ Peptides and proteins ⁃ macromolecules (kDa-range, including FITC-dextran models) ⁃ Vaccines and nucleic acids	⁃ Mechanism: LTRs and transient aqueous pores in SC lipid pathways. ⁃ Reversible Electroporation: generally mild, reversible barrier decrease ⁃ SC structure restoration reported after treatment. ⁃ Higher fields: irreversible EP associated with cell death and necrosis risk.	[[114], [115], [116], [117]]
6.	Magnetophoresis	⁃ Magnetic field strength (reported ∼5–300 mT) ⁃ magnet–skin distance ⁃ patch geometry/field distribution ⁃ exposure time (hours)	⁃ No physical penetration depth (no microchannels) ⁃ Enhancement is flux-based across SC into viable epidermis	⁃ 0 h direct barrier breaching (mechanistic work reports no modulation of SC permeability enhancement attributed mainly to magnetokinesis.	⁃ 0 h barrier resealing time (no structural barrier disruption reported in mechanistic studies).	⁃ Primarily small molecules/ions reported (e.g., lidocaine HCl, benzoic acid, salbutamol sulfate, terbutaline sulfate) ⁃ wearable example: ibuprofen patch	⁃ Biopsy histology is often not reported; safety is commonly assessed by skin reaction. ⁃ Example clinical use (48 h): irritation scale 0–3, 75% (18/24) score 0, mild redness mainly at the adhesive area.	[118,119]
7.	Thermal ablation	⁃ Surface temperature: 290–1035 °C (steam ejectate); 600–700 °C (heated filaments) ⁃ Pulse/exposure ⁃ Aperture geometry ⁃ pore density (pores/cm^2^)	⁃ Primary target: SC removal ⁃ Reported micropore depth: ∼13–21 μm (pulse 1.1–4 ms)	⁃ During pulse: μs–ms (single actuation) ⁃ Functional permeability increase: hours (commonly measured up to 12–48 h in diffusion studies)	⁃ Electrical resistance and impedance recovery reported within ∼12 h (in vivo)	⁃ Small molecules (e.g., sulforhodamine B model) ⁃ peptides/proteins (e.g., insulin; BSA model); ⁃ macromolecules (e.g., FD4 ∼4 kDa)	⁃ confirms selective SC removal with viable epidermis/dermis preserved under appropriate settings ⁃ micropore geometry and depth measurable (e.g., defined by mask windows)	[[120], [121], [122]]
8.	Laser ablation	⁃ Laser type/wavelength (commonly Er:YAG 2.94 μm) ⁃ Pulse energy/fluence ⁃ Pulse number ⁃ Fractional density/spot geometry ⁃ Exposure time	⁃ Micropore diameter: 150–200 μm ⁃ Lower energies can achieve SC-selective ablation	⁃ Typical array creation time: seconds ⁃ Reported barrier integrity loss window (TEWL): up to ∼6 h after AFXL in some studies	⁃ Recovery is protocol- and wear-dependent ⁃ TEWL may return toward baseline within ∼18–36 h in reported contexts ⁃ Microchannels often reseal within ∼1–2 days, with “complete” recovery reported by ∼3 days in longer-wear settings	⁃ Small molecules (e.g., lidocaine) ⁃ Broader reports include hydrophilic/lipophilic small molecules, proteins/antibodies, vaccines, and nanoparticles	⁃ Imaging/histology commonly shows well-defined cylindrical conduits. ⁃ Appropriate settings produce localised microchannels without deep tissue destruction, while higher settings increase thermal injury risk.	[[123], [124], [125]]
9.	Radio frequency ablation	⁃ Energy per microspot (e.g., ∼30 mj); ⁃ Microchannel density (e.g., ∼150–450 channels/cm^2^ depending on settings/study) ⁃ Electrode geometry/array design; pulse duration ⁃ Impedance/contact conditions (if feedback used)	⁃ Fractional microchannels extending into epidermis ⁃ Example pore depth ∼35 μm at ∼30 mj with minimal SC removal ⁃ Reported detectable delivery to deeper epidermal layers for some payloads	⁃ Immediate barrier disruption after treatment ⁃ baseline <8.5 g/m^2^·h, increased by > 20 g/m^2^·h post-RF (reported in porcine work)	⁃ Histology shows microchannels sealed at ∼24 h with new epidermis formation ⁃ Inflammation timeline: 4 h acute inflammatory cells; ⁃ 8 h granulation and epithelial regeneration	⁃ Peptides (kDa-range) ⁃ nucleic acids (e.g., siRNA ∼10–15 kDa) ⁃ macromolecule models (e.g., dextrans up to ∼40 kDa)	⁃ Microscopy and histology commonly confirm microchannel formation and depth ⁃ porcine histology reports early inflammation followed by regeneration and closure within ∼24 h (protocol dependent)	[126,127]

## 4 Drug delivery activation technologies

**Fig. 4:**
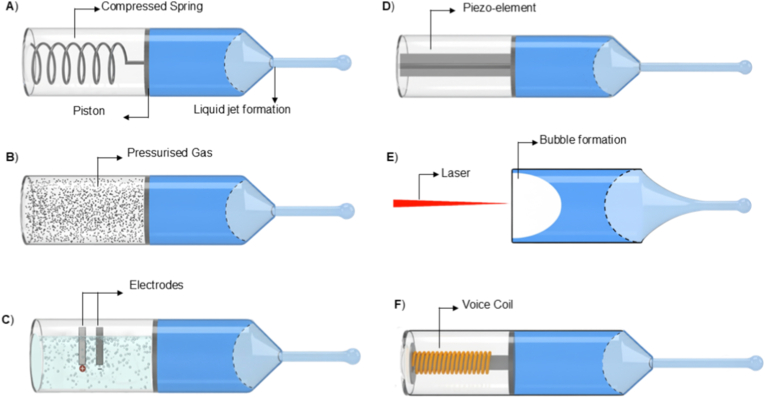
Schematic illustration of various activation mechanisms used in injector systems: A) spring-driven; B) gas-powered; C) electrochemical; D) piezoelectric; E) laser-induced; F) electromagnetic activations.

Building on the permeation-enhancement modalities discussed above, the next determinant of TNFDD performance is activation. Activation is responsible for initiating and controlling the release of the drug from its reservoir and directing it toward or through the skin, thereby playing a significant role in both timing and accuracy of delivery [128]. An activation mechanism is a key element of transdermal drug delivery systems. Activation is responsible for initiating and controlling the release of the drug from its reservoir and directing it toward or through the skin, thereby playing a significant role in both timing and accuracy of delivery [128]. Although TNFDD platforms vary widely based on their delivery modality, whether liquid jet, powder, or thermal poration, all rely on some form of mechanical, chemical, or electrical activation to initiate drug deployment. The activation mechanism of TNFDD enables the engine that delivers the payload at the right velocity, pressure, and dose. Thus, activation plays a dual role: enabling physical penetration and ensuring pharmacological precision [129]. The several types of activation mechanisms can be broadly classified as mechanical and non-mechanical systems, including spring-driven, gas-powered (pneumatic or compressed gas), electromagnetic, combustion-based, electrochemical, laser-induced, and piezoelectric approaches. Fig. 4 shows a visual representation of the different activations used in jet injector systems. This section will outline the fundamental principles behind each activation modality, discussing their integration with specific TNFDD platforms, and exploring parameters such as energy efficiency, size constraints, triggering methods, and delivery precision. Real-world examples will be presented to illustrate their application in both clinical and experimental contexts, alongside performance metrics.

### 4.1 Spring-driven activation technologies

Spring-driven actuators are a mechanically robust and energy-efficient solution for TNFDD. These systems have the ability to store and release mechanical energy without the reliance on electrical or chemical power sources, making them particularly attractive for decentralised, low-resource, and long-shelf-life applications [128]. The most commonly employed spring types are constant force and power springs, which are typically fabricated from high-tensile stainless steel alloys such as AISI 301 or EN 10088-3 X10CrNi18-8 [130]. These materials offer high elastic limits and fatigue resistance, which are essential for device longevity and reusability. The force output of constant force springs is governed by geometric and material parameters, with spring thickness (*t*) exerting a cubic influence on force (*F*): as described by:

(2) $$\begin{array}{c} F\propto \frac{E.\omega .t^{3}}{R} \end{array}$$

where *E* is the elastic modulus, *ω* the spring width, and *R* the coil radius. This relationship enables coarse tuning of jet velocity and penetration depth, supporting application-specific optimisation [130]. In practice, spring-powered injectors store energy in a compressed spring, which is then released to drive a piston and generate a high-velocity liquid jet. The jet velocity and impact pressure can be estimated as:

(3) $$\begin{array}{c} P=\frac{F}{A_{0}}=\frac{\rho v^{2}}{2} \end{array}$$

**Fig. 5:**
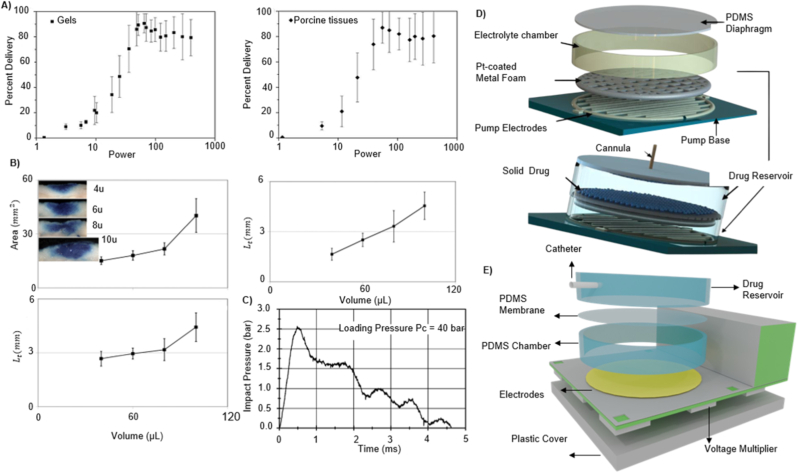
Performance characteristics of different activation mechanisms in TNFDD: A) Relationship between jet power and the percentage of fluid delivered into (a) gels and (b) porcine tissue using jet injection. Reprinted with permission from Ref. [132]; B) Injection of methylene blue into porcine skin showing area, depth, and distribution of delivered solution. Reprinted with permission from Ref. [133]; C) Representative measurement signal recorded from the impact plate under gas-driven activation, illustrating a maximum pressure of p_max_ = 2.57 bar. Reprinted with permission from Ref. [134]; D) Platinum-coated mesh electrolytic actuator for drug delivery, shown as an exploded diagram of components and the assembled system. Reprinted with permission from Ref. [135]; E) Exploded-view schematic of the electrolytic micropump, showing the electrochemical actuator and its major components [136].

Clinical and experimental applications demonstrated the practical utility of spring-driven systems. The Comfort-In™ device delivers high-velocity liquid jets (∼100–200 m/s) by employing a spring with minimal pain and pharmacokinetics comparable to subcutaneous injections [131]. Zeng et al. developed a similar system with adjustable micro-nozzles (0.17–0.50 mm) to deliver fluids into gels and porcine tissue analogues, confirming that spring activation alone can achieve precise, reproducible injections without electrical or pneumatic assistance [132]. Fig. 5A shows the relationship between jet power and the percentage of fluid delivered using a spring-actuated injector. Similarly, Schoubben et al. further characterised a spring-powered injector with an 80-μm micronozzle [133]. The force sensor analysis demonstrated that spring compression scales with injection volume, with larger doses producing higher piston forces and higher jet speeds (∼160–200 m/s). The resulting penetration depths were reproducible (8–14 mm in gels; 2–4 mm in porcine skin) and largely independent of fluid viscosity. As shown in Fig. 5B**,** histological sections of porcine skin confirm consistent deposition profiles across increasing doses, demonstrating the potential of spring-actuated injectors for precise transdermal administration. One of the main challenges in integrating spring actuators with the drug delivery system is their static nature, which might limit the dynamic control during injection [137]. Zhong et al. reported the spring dynamics and force profiles, enabling more predictable and consistent performance [138].

Spring activation remains inherently open-loop, lacking real-time feedback mechanisms that could adapt to patient-specific variables, such as skin resistance. Another challenge associated with spring-based systems is the gradual decline in force over extended stroke lengths, which affects injection consistency. This can be addressed through design strategies such as stiffer springs or mechanical compensation.

### 4.2 Gas-propelled activation technologies

Gas-driven systems utilise compressed gases, commonly CO_2_ or inert gases, stored at pressures ranging from several to tens of atmospheres [139]. The rapid release of the pressurised gas creates a steep pressure gradient, deforming or rupturing the pressure-sensitive diaphragm to propel the drug through a micro nozzle at very high velocity. The combination of optimal pressure magnitude and flow duration can produce a jet with ballistic or quasi-ballistic velocities, which is sufficient for dermal or subdermal needle-free injection [140]. Among gas-driven systems, supersonic jet injectors for powdered drug delivery represent a particularly demanding subclass. Ziegler et al. reported a needle-free powder injection device that utilises compressed gas to propel protein formulations through a nozzle at high velocity [134]. The device consists of a pressurised gas canister and a drug-loaded cassette; rupture of the cassette diaphragm releases the powder as a supersonic jet. The mechanical output of the activation, including impact forces generated during powder delivery, was characterised using an impact plate sensor (Fig. 5C). The measured pressure profile illustrates the rapid pressure rise and decay resulting from gas expansion and powder acceleration, providing a quantitative representation of the forces experienced by the payload during injection.

Gas activation technologies are not limited to compressed gas systems; some devices generate gas internally through the combustion of chemical propellants. These systems include a power source, either mechanical or electrical, that ignites the chemical compound. The resulting combustion rapidly produces gas, generating high pressure within the device. In line with this concept, Portaro et al. investigated a novel detonative combustion-based activation mechanism for needle-free liquid jet injection [141]. The controlled detonative combustion of a gaseous fuel oxidiser mixture (e.g. acetylene oxygen) generates a powerful shockwave. This detonation wave directly drives a piston, converting the chemical energy into mechanical work to pressurise the liquid drug chamber. Unlike conventional air-powered or electrically driven injectors, this method offers a significantly more powerful and tunable activation source.

Despite the mechanical effectiveness, gas-propelled devices have distinct design and operational challenges. Even though compressed gases offer higher energy density than conventional spring mechanisms by enabling more compact device architectures, this advantage is offset by limitations in storage stability and single-use design. Their reliance on gas containment necessitates specialised engineering, including reinforced housing and advanced sealing technologies. Gas-based injectors, particularly with prefilled drug cartridges, only have a shelf life of up to 2 or 3 years [142]. Beyond that, gas depletion or leakage may compromise the performance of the device. Alternatively, liquefied gases can be stored at ambient conditions as a potential solution. However, their sensitivity to temperature fluctuations will introduce variability in the activation dynamics and compromise delivery consistency.

### 4.3 Electrochemical activation technologies

Electrochemical activation is among the most widely used mechanisms after traditional mechanical activation. Unlike mechanical, this method operate by converting electrical energy into chemical energy via electrolysis [143]. Gas bubbles are generated through electrolysis in the electrolyte chamber, building up pressure that acts as the driving force to propel the drug, either through chamber expansion or controlled bursting [144]. Most electrochemical activation mechanisms in TNFDD systems employ electrolytic pumps to drive drug release. The system consists of a flat polydimethylsiloxane (PDMS) membrane, an electrolytic chamber, and a drug reservoir, with the PDMS membrane positioned between the two to actuate drug movement [145]. The reservoir will be integrated with the nozzle to facilitate the ejection of drugs at maximum velocity.

While designing an electrochemical actuator, the most critical aspect is electrode selection, as prolonged exposure to the electrolyte and repeated usage should not compromise device performance or drug stability [146]. Since the PDMS membrane is placed between the electrolytic chamber and the drug reservoir, it prevents direct contact and reduces the risk of electrochemical degradation of both the drug and the electrode. The activation cycle is governed by precise electrical control: power is applied until the membrane deflection reaches a predefined safe limit, after which it is withdrawn, allowing the pressure to dissipate.

Yi et al. reported a notable example of electrochemical activation, where platinum-coated nickel (Pt–Ni) metal foam serves as a catalytic reformer to enhance recombination of hydrogen and oxygen gases [135]. The system is built around a solid drug-in-reservoir (SDR) configuration (Fig. 5D), where drug release is mechanically driven by gas-induced membrane deformation. Dong et al. also demonstrated a PDMS membrane-based electrochemical micropump integrated with a miniaturised wireless power transfer (WPT) system, enabling battery-free, on-demand drug delivery [136]. Fig. 5E shows the schematic design of the electrolytic micropump. The device (20 × 14 × 8.2 mm^3^ in size) was actuated by low-voltage electrolysis powered by an RF-based WPT module operating between 0.8 and 1.3 V over distances up to 20 cm. As reported, the micropump delivered liquid continuously at flow rates ranging from 0.11 to 4.84 μL/min, achieving a total volume of 100.8 μL. This work serves as proof of concept for electrochemical activation, demonstrating its relationship with wireless energy systems and miniaturised drug delivery platforms. Although electrochemical actuators offer numerous advantages, their shelf life is extremely limited. Due to electrolyte degradation and electrode corrosion, these devices have a shelf life of only 3–5 years, which significantly impacts their marketability and limits large-scale public deployment.

### 4.4 Piezoelectric activation technologies

Piezoelectric activation offers an alternative to conventional high-force mechanisms, which offer greater controllability and can be electronically configured [147]. Piezoelectric materials have a crystalline lattice with a non-centrosymmetric structure, which gives rise to a permanent electric dipole. When an electric field is applied, the dipoles within the crystal align with the field, inducing stress in the material and resulting in mechanical displacement (inverse piezoelectric effect) [148]. However, the generated mechanical stress is relatively low in absolute terms. This limitation is addressed by implementing a multilayer configuration, which actively drives the liquid out of the device at higher velocities. Lead zirconate titanate (PZT) is a widely used piezoelectric material and is non-centrosymmetric, exhibits strong electromechanical coupling, and has ferroelectric and ionic polarisation, making it well-suited for actuators. The performance of PZT can be enhanced by doping with elements such as barium, lanthanum, and niobium, and by adjusting the magnitude of strain and response time to achieve optimal drug delivery [149].

Piezoelectric actuators are in various devices, including micropumps, microvalves, and micromotors. To deliver microliter-scale volumes, micropumps are typically employed, either by deflecting a membrane or using resonant vibration [150]. Microvalves, on the other hand, are used to control fluid flow through small channels, whereas micromotors are preferred for linear and rotational movement of a plunger [151]. Meanwhile, piezoelectric transducers are commonly used in ultrasound-assisted delivery to enhance skin permeability. Trimzi et al. reported a system employing a multilayer PZT actuator for TNFDDS [152]. They demonstrated impulse forces of up to 3500 N s, sufficient to drive a 10-μL volume of drug through the skin with minimal tissue disruption. Despite their precision and responsiveness, piezoelectric actuators are inherently limited in displacement volume, typically operating within the nanolitre-to-microliter range. This restricts their use in applications requiring high-volume drug administration. To address this limitation, large-stroke actuators such as voice coils have been explored. In one example, a voice coil-powered injector delivered up to 1.3 mL of fluid with dynamic pressure control, achieving ±1% accuracy in ex-vivo tissue. The system allowed programmable jet velocity during the injection pulse, enabling real-time control of penetration depth and dose features hard to achieve with commercial piezoelectric systems [153]. Despite all the advantages, as mentioned, the low stress generated allows only a small volume of liquid to be displaced, limiting their use in high-dose applications. Delivering higher volumes requires repeated cycles, which cause material fatigue over time and reduce actuator performance.

### 4.5 Laser-induced activation technologies

Laser-induced activation leverages localised thermocavitation to generate high-speed microjets. Unlike laser-assisted ablation, which targets the skin directly, this approach induces cavitation at the interface between the device and the drug fluid. Upon focused laser irradiation, rapid thermal expansion and a simultaneous pressure drop create a transient vapour bubble [154]. The dynamics of these bubbles can be described by the Rayleigh–Plesset equation, which relates the temporal change in bubble radius to the surrounding pressure field, surface tension, and viscous forces:

(4) $$\begin{array}{c} \rho \left(R\frac{d^{2}R}{dt^{2}}+\frac{3}{2}\;{\left(\frac{dR}{dt}\right)}^{2}\right)=P_{0}-P_{V}-P\left(t\right)-\frac{2\sigma }{R}-\frac{4\mu }{R}\frac{dR}{dt} \end{array}$$

where *R* is the radius of the bubble, $\frac{dR}{dt}$ is the rate of change of the radius (velocity of the bubble wall), $\frac{d^{2}R}{d\;t^{2}}$ is the acceleration of the bubble wall, ρ is the density of the liquid, $P_{0}$ is the ambient pressure, $P_{V}$ is the vapour pressure inside the bubble, *P*(*t*) is the time-dependent external pressure applied to the bubble (which could include laser-induced effects), σ is the surface tension, and μ is the viscosity of the liquid [155]. The collapse of this bubble generates a sharp impulse that propels a controlled volume of drug through a micro-nozzle, forming a high-velocity jet capable of penetrating the skin without needles. This activation method is particularly compatible with miniaturised jet injectors, offering electronically tuneable, non-contact delivery with high spatial and temporal precision [156]. Unlike other activation methods, laser activation has no mechanical moving parts and relies solely on optical energy, making it easier to integrate into wearable devices and needle-free drug delivery systems.

**Fig. 6:**
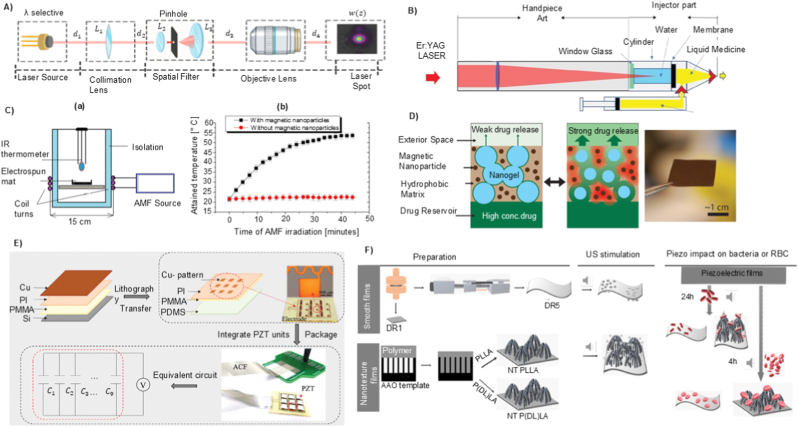
Performance characteristics of different activation mechanisms in TNFDD: A) Schematic of the optical setup for laser-induced injection. Reprinted with permission from Ref. [155]; B) Mechanism of the laser-induced needle-free microjet injector. Reprinted with permission from Ref. [157]; C)Alternating magnetic field–induced heating of nanofibers with embedded magnetic nanoparticles: (a) experimental setup (b) temperature evolution in the nanofiber patch [158].; D); Composite membrane containing nanogel particles (blue), iron oxide nanoparticles (dark brown), and ethyl cellulose matrix (light brown); magnetic field–induced heating causes nanogel shrinkage and drug release (green). Reprinted with permission from Ref. [159]; E) Fabrication and design of the flexible ultrasonic patch: (a) PMMA, PI, and Cu layers spin-coated on Si substrate; (b) lithography, etching, and transfer printing to form the flexible circuit; (c) integration of PZT-4 piezoelectric units and packaging with a ≈200 μm hydrogel patch. Reprinted with permission from Ref. [160]; F) Preparation of smooth piezoelectric (DR5) and non-piezoelectric (DR1) and nanotextured piezoelectric (NT PLLA) and non-piezoelectric (NT P(DL)LA) polymer films, showing expected response to ultrasound stimulation [161].

Taha et al. reported an example prototype is the laser-induced microjet injector, utilising a laser-generated vapour bubble within a microfluidic chamber to eject microliter-order drug doses at jet velocities [155]. Fig. 6A depicts the optical setup used for laser-induced injection, showing the geometry and alignment of the laser and target components. In another study, Oh et al. reported a laser-driven needle-free jet injector (Mirajet) for transdermal delivery of a poly-dl-lactic acid (PDLA) filler. The system employed Er: YAG laser pulses (70–90 mJ, 30 Hz) focused into a sealed microfluidic chamber terminating in a 200-μm-diameter nozzle [157]. Fig. 6B illustrates the mechanism of the laser-induced needle-free microjet injector. Absorption of the laser energy by the chamber wall produces rapid localised vaporisation, generating a cavitation bubble whose collapse induces a transient pressure spike. This drives the fluid bolus through the nozzle at jet velocities sufficient to penetrate the epidermis and reach the papillary dermis (∼300 μm) without mechanical needles.

From an engineering perspective, laser activation is an optofluidic activation, where photothermal energy is converted into a mechanical impulse for drug delivery. However, small deviations can produce asymmetric bubble collapse and off-axis jets, which increase the mechanical stress on chamber walls [162]. Moreover, the requirement of a high-power laser source restricts the miniaturisation and the cost efficiency. Future developments could involve the integration of solid-state micro-lasers, monolithic chamber fabrication, and active alignment feedback systems to enhance robustness and scalability.

### 4.6 Electromagnetic activation technologies

Electromagnetic activation, similar to electrochemical methods, enables needle-free drug delivery by utilising magnetic energy to propel or assist the transport of the drug [163]. In particular, Lorentz-force–based systems allow dynamic control of jet velocity and injection depth through feedback-regulated electromagnetic actuation [164]. These systems operate on the principle of the Lorentz force, where a current-carrying coil in a magnetic field generates an axial force. This force drives a linear actuator, often similar in design to voice coil motors, to propel the drug ampoule and eject fluid at high pressure. A notable advancement in this domain is the system developed by Portaro et al., which employs a three-phase linear motor with fixed coils and multiple permanent magnets [141]. This architecture allows real-time control over plunger velocity and injection pressure, including pulsed delivery modes. Taberner et al. developed a servo-controlled jet injector that integrates a handheld unit with a custom linear Lorentz-force motor (peak force of ∼200 N) and a real-time FPGA-based controller [165]. The motor drives a piston through a 220-μm nozzle, producing jet velocities up to 200 m/s, while hybrid feed-forward/PI feedback control (64 kHz) enables precise trajectory tracking. The cRIO controller, manages the operation of the handheld injector and the linear Lorentz-force motor.

Furthermore, magnetically responsive nanofiber patches have been engineered to achieve remote-controlled drug release [158]. These patches are fabricated via electrospinning of hydrophobic polycaprolactone (PCL) fibres embedded with uniformly distributed magnetic nanoparticles, creating a flexible and skin-conformal matrix. Upon exposure to an alternating magnetic field, the nanoparticles generate localised heat through magnetic hyperthermia, which raises the temperature of the PCL fibres above their melting point (≈45–52 °C). This thermal transition triggers structural relaxation of the fibres, facilitating the controlled release of encapsulated drugs such as tazarotene. Fig. 6C shows the experimental setup and temperature evolution of the magnetic nanofiber patch under alternating magnetic field exposure, illustrating both the heating mechanism and the resulting thermal response of the system.

Hoare et al. reported a composite membrane system designed to achieve precise, on-demand control of therapeutic release [159]. These membranes integrate superparamagnetic iron oxide nanoparticles as localised heat sources and temperature-sensitive PNIPAm-based nanogels embedded within an ethylcellulose matrix. Upon exposure to an oscillating magnetic field, the nanoparticles generate heat, inducing a reversible phase transition in the nanogels that opens transport pathways for drug molecules from a reservoir. The membrane (Fig. 6D) exhibits a uniform distribution of nanoparticles and nanogels within the polymer matrix. Magnetic heating drives nanogel shrinkage, transiently opening diffusion channels for controlled release. By tuning nanogel composition, membrane thickness, and nanoparticle loading, the release rate and on/off switching behaviour can be rationally controlled for drugs with varying molecular weights and charges. The system demonstrates zero-order release kinetics during the “on” state, high reproducibility across cycles and devices, and flexibility in adjusting drug dosing through both material engineering and parameters of magnetic field.

Despite their technical advantages, electromagnetic actuators present several challenges that limit their widespread application. The integration of high-speed linear motors and closed-loop control systems increases the complexity and cost. While compact designs are emerging, many Lorentz-force based systems still require substantial power input and bulky components, limiting their suitability for wearable or portable applications. While considering magnetic activation, it is notable that most reported devices are implantable rather than conventional transdermal systems; they can still be categorised under needle-free drug delivery due to their non-invasive drug release.

### 4.7 Ultrasound-based activation

Ultrasound has been investigated as a triggering mechanism in TNFDD systems, extending beyond its conventional role in physical permeation enhancement. This approach combines mechanical and mild thermal effects to trigger or tune drug release [71]. In many designs, microbubbles act as an acoustic transducer when they are exposed to ultrasound; they oscillate and cavitate, creating local pressure pulses and small temperature gradients. These coupled effects can generate the impulses needed to propel the drug, rupture carriers, or disturb the matrix enough to release its payload. Huang et al. demonstrated this concept using an ultrasound-responsive hydrogel patch designed for controlled release and improved penetration through an acoustic–mechanical mechanism [166]. In their system, diclofenac sodium–loaded PEG–PLGA microcapsules were embedded within a crosslinked four-arm PEG hydrogel. When ultrasound was applied (2 W cm^−2^), the alternating pressure field drove volumetric oscillations and progressive mechanical fatigue of the microcapsules, ultimately rupturing the capsule walls and producing a burst release of diclofenac. At the same time, cavitation and microstreaming at the hydrogel–skin interface transiently disrupted SC lipid organisation, enabling faster transdermal diffusion. The patch architecture also matters here: the uniform distribution of ∼3.5 μm microcapsules within an elastic, skin-conformal PEG matrix helped transmit acoustic energy more evenly, promoted homogeneous stress propagation, and reduced energy loss during ultrasound delivery.

Beyond microbubble-assisted systems, some wearable devices use piezoelectric materials to translate ultrasound into a direct actuation signal. In contrast to conventional sonophoresis, where ultrasound mainly increases permeability through cavitation, piezoelectric ultrasound platforms can convert acoustic input into an electrical output via the direct piezoelectric effect, which then triggers or modulates release. For instance, Lyu et al. developed a flexible ultrasonic patch (FUSP) for chronic wound healing in diabetic models [160]. The design embeds micro piezoelectric ceramic units (PZT-4) in a soft PDMS substrate using an island–bridge serpentine layout, allowing the patch to remain mechanically flexible while maintaining close contact with the skin. Fig. 6E shows the fabrication of the patch, flexible circuit design, and integration of PZT-4 ultrasonic units. During low-intensity ultrasound activation (≈2.2 MHz, <50 mW cm^−2^), the device generates acoustic stimulation that reactivates senescent fibroblasts through the Ca^2+^/CaMKII–Tiam1–Rac1-GTP signalling pathway, promoting angiogenesis and tissue regeneration. Similarly, Gazvoda et al. reported an antimicrobial patch based on piezoelectricity [161]. The device employs a poly-L-lactic acid (PLLA) film, engineered with smooth or nanotextured surfaces, to facilitate ultrasound-triggered drug delivery. When exposed to ultrasound (37–80 kHz), mechanical vibrations deform the piezoelectric film, generating localised electric charges through the shear piezoelectric effect. These charges induce controlled permeabilisation of the drug reservoir interface and facilitate the release of the loaded drug. Nanotextured films enhance this effect by increasing surface area and mechanical responsiveness, allowing for more efficient drug release under the same conditions. Fig. 6F shows the fabrication of smooth (DR1, DR5) and nanotextured (NT P(DL)LA, NT PLLA) piezoelectric and non-piezoelectric polymer films, along with their piezoelectric response and biological interactions.

### 4.8 Optical-based activation

Optical energy is also employed as an activation method in drug delivery as a safer alternative to laser activation [167]. Typically, near infrared (NIR) or visible light sources are employed to trigger drug release from the device through photothermal, photochemical, or photoacoustic mechanisms [168]. Upon absorbing optical energy, these devices generate localised heating that promotes gel diffusion by inducing mechanical stress or triggering chemical transformations within the system. Because this activation method requires no batteries or electrical components, light-based activation is primarily used in transdermal patches rather than in wearable devices [169,170]. Light-triggered drug delivery devices often face challenges in efficiently converting light energy into a form capable of driving drug release. Photo-responsive systems typically work by embedding light-sensitive “converters” inside soft matrices such as hydrogels. Materials like upconversion nanoparticles (UCNPs), gold nanorods, or carbon-based absorbers can absorb light and transform it into a local trigger, most often heat, but sometimes a chemical change. This local response can soften or disrupt the matrix, create pressure build-up or phase changes, and ultimately act as the activation step that initiates drug release or enhances transport across the stratum corneum.

For example, Dhal et al. reported an NIR-triggered approach in which lithium-doped UCNPs were incorporated into an oleogel matrix [171]. Under NIR irradiation, photothermal conversion softened the oleogel microstructure, lowered its viscosity, and promoted diffusion across the stratum corneum. In a different design, Li et al. developed flexible photothermal patches using Kapton substrates patterned with gold nanoholes and coated with reduced graphene oxide to improve light absorption [172]. The gold nanohole arrays, fabricated via colloidal lithography with polystyrene spheres—support plasmonic resonances that efficiently convert 980 nm light into heat. In practice, this creates a thin, tunable heating interface, where the photothermal output can be adjusted simply by changing laser power and exposure duration.

More advanced wearable systems go a step further by linking light-triggered release with real-time sensing for truly on-demand therapy. Pang et al., for instance, developed a flexible, electronics-integrated wound dressing that continuously monitors wound temperature and releases antibiotics only when infection-related hyperthermia is detected [173]. The device uses a UV-responsive hydrogel, where the antibiotic is tethered through a UV-cleavable linker. When the embedded 365 nm UV-LEDs are switched on, the linker breaks and the drug is released directly at the wound site, helping to suppress bacterial growth.

In summary, activation technologies define how efficiently a TNFDD system converts energy into a reproducible therapeutic event. Across these activation families, several recurring design trade-offs emerge, including energy density and portability, open-loop simplicity and feedback-enabled control, rapid response speed and thermal or electrochemical side effects, and long-term stability and cost as well as manufacturability. These considerations ultimately determine whether a given permeation strategy can be translated into a compact and reliable device. With the permeation mechanisms and their activations established, the next section synthesises these design variables into a comparative framework, highlighting performance metrics and practical constraints that govern platform selection and clinical translation.

## 5 Recent developments in transdermal needle-free drug delivery systems

**Fig. 7:**
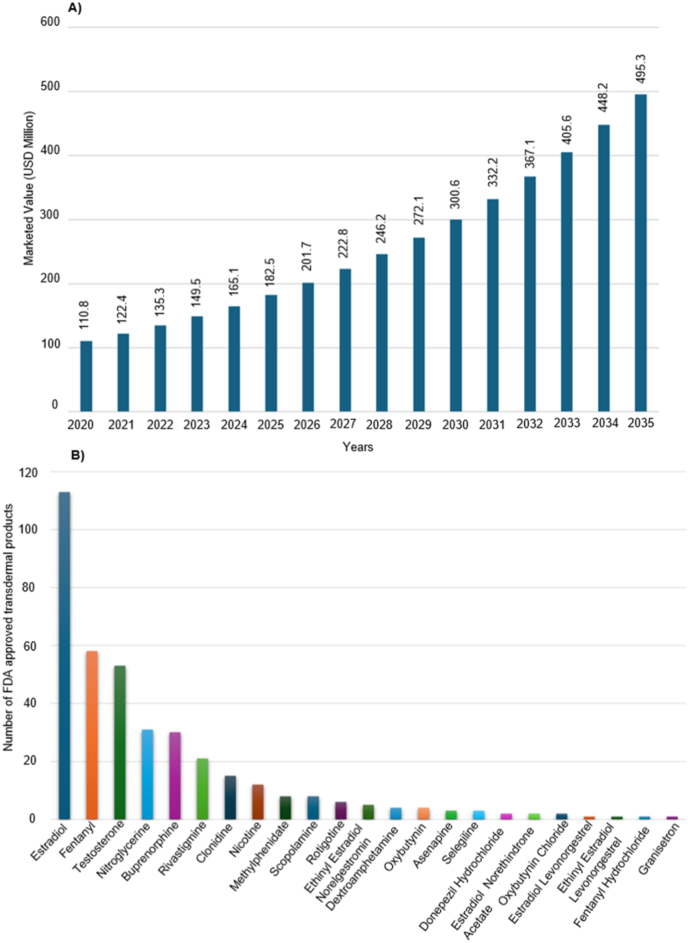
(A) Predicted growth in the market value of needle-free transdermal drug-delivery devices from 2020 to 2035 (USD million). (B) Drug-wise distribution of FDA-approved transdermal therapies and the number of commercially available transdermal products, illustrating the expanding adoption and continued relevance of transdermal delivery.

The advancement of various skin permeation technologies and activation mechanisms has significantly expanded the accessibility of transdermal drug delivery, stimulating growing research interest in this field. Recent market analyses support this, reporting a projected compound annual growth rate (CAGR) of 10.5% between 2025 and 2035 [174]. Fig. 7A presents a graphical representation of the market analysis for needle-free transdermal drug-delivery devices. The current industrial trend is summarised in Table 2, which lists available needle-free transdermal drug delivery systems on the market that employ a range of activation technologies. The devices available on the market primarily utilise skin permeation mechanisms, including high-speed jets, iontophoresis, and ultrasound, while most of their activation methods rely on spring-based or compressed-gas systems to deliver drugs. Most of the devices on the market are designed to deliver insulin, lidocaine, and certain vaccines, targeting applications such as diabetes management, immunisation, cosmetic dermatology, and pain management [82]. Moreover, several clinical trials are currently investigating the mechanisms and device platforms discussed in this paper, underscoring the relevance and growing clinical momentum of transdermal needle-free drug delivery.

**Table 2:** Overview of selected commercially available needle-free drug delivery devices, highlighting the companies involved, actuation technologies, mechanisms of physical penetration, active drug formulations, and associated clinical indications.

Sl No	Manufacturing Company	Brand name	Activation Technology	Skin penetration enhancement techniques	Active Drug	Condition/disease	Ref
1.	West pharmaceutical	Smartdose®	Battery-powered motor system	Jet injection	Biologics (e.g., monoclonal antibodies)	Chronic conditions	175
2.	Antares Pharma, Inc	Medi-Jector VISION®	Spring-powered		Insulin	Diabetes	176
3.	Health/Comfort-in	Comfort-in™			Insulin, lidocaine, testosterone, B12, botulinum toxin, hyaluronic acid	Diabetes, anaesthesia, and hormone therapy	177
4.	Pro-care management	Vitajet™ 3			Insulin	Diabetes	178
5.	Visionary Medical Products Corporation (VMPC)	Penjet®	Compressed gas propulsion		Various (prefilled single dose)	Mass immunization	179
6.	National medical products	J-tip®			Lidocaine (xylocaine)	Local anaesthesia for IV starts, blood draws	180
7.	Crossject	ZENEO®			Epinephrine, Midazolam, Hydrocortisone, Sumatriptan, Naloxone	Anaphylaxis, seizures, adrenal crisis, migraine, opioid overdose	181
8.	Bioject Medical Technologies Inc.	Biojector® 2000			Vaccines, various injectables	Immunisation, general injection use	182
9.	Sontra Medical Corp	Sonoprep	Low-frequency ultrasound	Sonophoresis	Lidocaine	Local anaesthesia	183
10.	Alza & crescendo	E-trans fentanyl	Electrochemical	Iontophoresis	Fenatyl	Acute pain	184
11.	Mdsc labs	Wrinklemd®			Hyaluronic acid (ha), peptides	Skin ageing, wrinkles	185

To complement the commercial landscape, Table 3 offers an overview of the current status of needle-free transdermal and skin-permeation–assisted technologies in clinical translation. It summarises the clinical trials on different payload types and study designs, based on records from the ClinicalTrials.gov database. In addition to that, the table highlights how each technology is being positioned clinically, the trial phase, and practical considerations that may influence dosing feasibility and patient adherence. The U.S. Food and Drug Administration (FDA) has already approved numerous drugs for conventional transdermal delivery, underscoring both the established clinical value of this route and the need to demonstrate reliable performance and safety. Fig. 7B provides a detailed breakdown of FDA-approved transdermally administered drugs and the corresponding number of marketed transdermal products available for each drug. This distribution helps identify drug candidates with strong established compatibility and clinical precedent, making them promising targets for needle-free transdermal delivery approaches. Numerous patents have also been filed for transdermal needle-free drug delivery over the years, reflecting the growing demand for these devices [128]. Table 4 lists some of the engineering designs patented under NFDD that use the activation technologies discussed in this paper.

**Table 3:** List of ongoing clinical trials investigating the needle-free transdermal drug delivery devices and technologies (data accessed on January 05, 2026).

Sl No.	Name of the trial	Technology	Device	Indication	Payload	Phase	Status	Trial ID	Notes	Ref.
1.	J-Tip Use for Paracentesis in Adults with Liver Cirrhosis and Ascites	Needle-free jet injection	J-Tip	Pain/anxiety reduction during bedside paracentesis in adults with cirrhosis and ascites	Lidocaine 1%	Not applicable	Recruiting	NCT06996379	A randomised, open-label, parallel study that compares intradermal lidocaine delivered by the J-Tip device with intradermal lidocaine delivered by a 25-gauge needle, while both groups still receive deeper subcutaneous lidocaine by a 22-gauge needle as standard care.	186
2.	Safety, reactogenicity and immunogenicity of a Venezuelan Equine Encephalitis DNA vaccine candidate administered by jet injection	Needle-free jet injection	PharmaJet Stratis (IM) and Tropis (ID)	Safety, reactogenicity and immunogenicity of the VEE NDA vaccine in healthy adults.	Venezuelan Equine Encephalitis (VEE) DNA vaccine	Phase 1	Active, not recruiting	NCT06002503	A randomised, observer-blind study that compares immunisation delivered by intramuscular jet injection with immunisation delivered by intradermal jet injection using the specified PharmaJet platforms.	187
3.	mRNA COVID-19 vaccine immune response comparisons using different delivery routes	Needle-free jet injection vs conventional IM injection	PharmaJet Tropis (ID) vs needle & syringe (IM)	Immune-response comparison for mRNA COVID-19 vaccine delivery routes in healthy volunteers	COMIRNATY® (mRNA COVID-19 vaccine)	Phase 2	Not yet recruiting	NCT06919796	This study compares the immunologic response produced by intradermal delivery using a needle-free jet injector with the response produced by conventional intramuscular delivery using a needle and syringe.	188
4.	Efficacy of iontophoresis in treating Lateral Epicondylitis Patients	Iontophoresis	Iontophoresis system (brand not specified)	Lateral epicondylitis (tennis elbow)	Magnesium sulfate (iontophoretic delivery)	Phase 1	Recruiting	NCT06578000	A randomised, double-blind trial in which the intervention arm receives magnesium sulfate iontophoresis in addition to conventional therapy, and the comparator arm receives conventional therapy alone.	189
5.	Acetylcholine iontophoresis as a new challenge with Type 2 diabetic peripheral neuropathy: A possible new therapy	Iontophoresis	Iontophoresis device/electrodes (brand not specified)	Type 2 diabetes with peripheral neuropathy	Acetylcholine (ACh)	Not applicable	Recruiting	NCT06219590	A randomised, parallel study that compares acetylcholine iontophoresis with a sham procedure and includes outcomes related to nitric oxide measures, neurophysiology testing, and pain.	190
6.	Neostigmine and Glycopyrrolate by iontophoresis to induce bowel evacuation	Iontophoresis	I-Box (Dynatronics; wired ION system)	Neurogenic bowel in spinal cord injury (constipation/faecal incontinence)	Neostigmine + Glycopyrrolate	Phase 3	Active, not recruiting	NCT06351995	The Study administers neostigmine and glycopyrrolate through the skin using a wired iontophoresis system, evaluates bowel-evacuation endpoints, and incorporates pharmacokinetic sampling while titrating the neostigmine dose to determine a lower effective dose.	191
6.	Sustained Acoustic Medicine (SAM) for symptomatic treatment of pain related to bone fracture	Ultrasound-mediated transdermal delivery (LITUS/sonophoresis-adjacent)	Wearable SAM/LITUS device and diclofenac patch	Bone fracture-related pain	Diclofenac 2.5% patch (used with SAM device)	Phase 1	Recruiting	NCT05883241	The study evaluates daily long-duration low-intensity therapeutic ultrasound applied at 3 MHz and 0.132 W/cm^2^ for up to 4 h per day over 12 weeks while diclofenac is delivered from a topical patch during treatment.	192
7.	Effect of insulin therapy by ultrasonography in wound healing of chronic diabetic patients	Sonophoresis	Therapeutic ultrasound device (brand not specified) and hyperpolarised light therapy (adjunct; in both arms)	Chronic diabetic wounds (wound healing in diabetes mellitus)	Insulin (topical/transdermal)	Not Applicable	Not yet recruiting	NCT06708975	A randomised two-arm study in which one group receives insulin delivered by phonophoresis together with hyperpolarised light therapy and standard wound care, while the comparator group receives hyperpolarised light therapy and standard wound care without insulin phonophoresis.	193
8.	Investigation of laser-assisted drug delivery of NanoDOX®	Laser ablation	CO_2_ Ablative Fractional Laser and topical hydrogel	Wounds/wound healing after ablative laser	NanoDOX® Hydrogel (1% doxycycline)	Phase 2	Active, not recruiting	NCT05411484	A self-controlled, single-site study in which an ablative fractional carbon dioxide laser is applied first and NanoDOX hydrogel is then applied topically, with serial skin biopsies used to quantify drug uptake depth and assess wound healing.	194
9.	Treatment of hypopigmented scars with bimatoprost	Laser-assisted drug delivery (LADD)	Fractional ablative CO_2_ laser (FLSR)	Hypopigmented burn scars/hypertrophic scar hypopigmentation	Bimatoprost (vs saline vehicle control)	Phase 2	Recruiting	NCT06122090	This within-patient controlled trial randomises two scars within the same participant so that both scars receive fractional ablative carbon dioxide laser treatment while only one scar receives bimatoprost delivered through the laser channels and the other scar receives saline delivered through the laser channels.	195

**Table 4:** Selected patents on needle-free drug delivery devices from academic and industrial sectors, illustrating key functional advancements and actuation mechanisms shaping the future landscape of NFDD systems.

Patent Number	Device name	Assignee	Function	Activation Mechanism	Ref
US10326347B2 (June 18, 2019)	Controlled Needle-Free Transport	Massachusetts Institute of Technology, Cambridge, MA (US)	Provides precise, programmable jet injection via pulsed magnetic control	Electromagnetic (Lorentz force)	196
US20220040410A1 (February 10, 2022)	Needle-Free Intradermal Injection Device	Pharmjet Inc., Golden, CO (US)	Designed for simple, spring-actuated intradermal drug or vaccine delivery	Spring Powered	197
US20230025722A1 (January 26, 2023)	Needle-Free Injector	Pharmjet Inc., Golden, CO (US)	Compact, reusable jet injector platform optimised for safety and ease of use	Spring Powered	198
US11571519B2 (February 7, 2023)	Bi-directional Motion of a Lorentz-Force Actuated Needle-Free Injector (NFI)	Massachusetts Institute of Technology, Cambridge, MA (US)	Enables programmable forward and reverse motion for advanced control of drug injection depth	Electromagnetic	199

### 5.1 Jet injectors

Although research on jet injection is actively ongoing, the number of available devices remains limited, with only a handful currently commercialised. Notably, the use of jet injectors for DNA vaccine delivery has attracted considerable attention, given the immunological richness of the dermal layer. One of these developments is the pyro-drive jet injector developed by Sonoda et al., which employs bi-phasic pyrotechnic compounds to generate high-velocity jets [200]. This high-energy kinematics provides additional dispersion of DNA into the dermis and has been correlated with increased transfection efficiency and immune stimulation. However, although such immunological benefits are present, utilisation of controlled explosive materials involves significant engineering and regulatory challenges. The use of pyrotechnics may raise concerns around device safety and manufacturing standardisation. In contrast, Trimzi et al. developed a mechanically controlled, compressed air-powered jet injector using a hydro-pneumatic system [201]. Their device features dual independent circuits, pneumatic for driving force and hydraulic for volume control. The system goes through activation, pressurisation, suction, and recharging phases through a pilot-operated 3/2 pneumatic valve, enabling automatic operation without electronic control. While this design elegantly achieves self-cycling delivery and precise volumetric control (0.2–0.5 mL), it relies on complex fluidic coordination and precise timing that increase dead volume and maintenance needs. Such intricacy poses major barriers to miniaturisation and portability in wearable or point-of-care settings. Replacing the pneumatic timing network with MEMS-based electro-pneumatic microvalves and embedding pressure and displacement microsensors for closed-loop feedback could address the limitations.

**Fig. 8:**
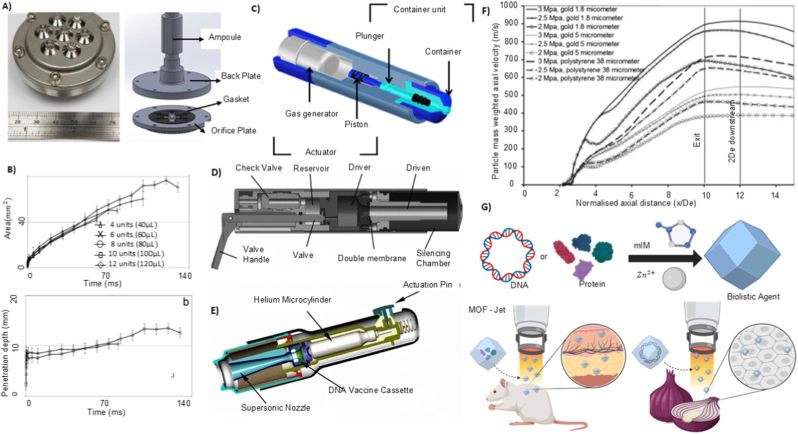
Performance characteristics of Jet Injectors in TNFDD: A) Physical prototype (left) and CAD rendering (right) of the seven-orifice jet injection nozzle [202].; B) Injection performance of the spring-actuated micronozzle injector: (a) Area expansion profiles for 40–120 μL methylene blue, (b) Penetration depth profiles for 80 and 120 μL injections. Reprinted with permission from Ref. [133]; C) Schematic diagram of Actranza™ pyro-drive jet injector. Reprinted with permission from Ref. [203]; D) Cross-sectional view of the reusable, needle-free biolistic intradermal injector. Reprinted with permission from Ref. [204]; E) Schematic of a commercially viable hand-held injector using supersonic nozzles for biolistic drug delivery systems. Reprinted with permission from Ref. [205]; F) Particle mass-weighted average axial velocity as a function of normalised axial distance for different particle types (gold, polystyrene) and driver pressures (2, 2.5, 3 MPa). Reprinted with permission from Ref. [46]; G) Schematic illustration of biomimetic mineralisation and biolistic delivery using the MOF-Jet [206].

A persistent challenge in jet injection remains the limited injectable volume. To address this, McKeage et al. developed a multi-orifice injection system. By parallelising up to seven nozzles, their system successfully delivered up to 2 mL of drug into ex vivo porcine tissue in under 0.15 s [202]. Their use of computational fluid dynamics (CFD) modelling and experimental data effectively supported the consistency of jet characteristics across orifices**.** Fig. 8A shows the physical prototype of the multi-orifice nozzle design and a 3D-rendered exploded view of the nozzle-ampoule, which is used to fire all jets consistently at ∼140 m/s. However, while the approach demonstrates scalability in volume, this approach introduces some additional engineering concerns. Synchronising multiple jets requires extremely tight tolerances in nozzle diameter, chamber pressure, and activation timing; any deviation can cause uneven penetration depth or jet deflection. Furthermore, distributing the driving pressure across several microchannels increases dead volume and viscous losses, reducing overall energy efficiency. This could be addressed employing precision-milled sapphire or silicon nozzles (<100 μm in diameter) combined with additive-manufactured manifolds and integrated pressure-balancing microvalves to minimise flow asymmetry and improve synchronisation.

In contrast to such high-power, multi-orifice systems, later approaches explored mechanically driven injectors to reduce power dependence and design complexity. Schoubben et al. presented a spring-actuated micronozzle injector designed for minimal power dependence [133]. Their use of polyacrylamide gels and porcine models confirmed a correlation between jet force and penetration. As shown in Fig. 8B, the injection area scaled predictably with volume (40–120 μL), and penetration depth increased modestly with higher doses. Meanwhile, Miyazaki et al. refined the pyro-drive jet injection concept through the Actranza™ system, which employs dual explosive agents with staggered burn rates to achieve fine-tuned modulation of jet pressure and penetration depth [203]. The device schematic shown in Fig. 8C illustrates the actuator and container unit. While this architecture demonstrates improved controllability over conventional injectors, its reliance on pyrotechnic components raises potential safety and acceptance challenges for clinical translation. Nevertheless, its compact design and adjustable pressure profile make it an attractive candidate for preclinical drug discovery applications.

In addition to innovations in injector mechanisms, several studies investigated the impact of injection parameters on the performance of liquid jet delivery, particularly for nanoparticle-based formulations. Park et al. investigated the influence of particle size, injection pressure, and syringe orifice diameter on transdermal nanoparticle delivery [207]. In this study, simulation and experimental validation demonstrated that as the nanoparticle size increased, from 45 nm to 452 nm, the penetration depth and channel diameter decreased, while lateral dispersion increased. Similarly, larger orifice diameters produced deeper and wider penetration, whereas higher injection pressures paradoxically decreased channel diameter and overall dispersion. These findings provide crucial insights for tailoring jet injector parameters depending on therapeutic payload characteristics. Rather than presenting a new device, the study underscores the importance of precise injector configuration to optimise delivery efficiency for nano formulations, especially in the context of transdermal and dermatological applications.

A major technical concern in jet injection lies in the high shear forces generated as formulations are expelled through micron-scale nozzles at high velocity. Although the residence time is low, such force can damage the structural integrity of biologics, proteins, and vaccines [208]. Registration precision, the absolute alignment of the nozzle tip within the skin, is another challenge. Under high pressure, even a slight slip of the injector can cause linear skin laceration, especially with small-diameter orifices [209]. This risk can be minimised by designing systems that are actuated upon skin contact, eliminating the need for a separate manual trigger and also completing the injection before human reaction time (100 ms) [39]. Jet injection also operates within a narrow performance window. Penetration and reliable delivery depend strongly on jet speed, typically near ∼100 m/s, and small shifts can cause under- or over-penetration [16]. In controlled large-volume studies, penetration typically required jet speeds around 80–100 m/s, while consistent delivery of a 1 mL bolus was observed when the jet speed reached approximately 130 m/s or higher. Jet geometry further influences efficiency, with classic data showing optimal performance near ∼150 μm nozzle diameters at high velocities [34]. As reported earlier, higher volumes can be engineered through parallelisation, but this approach also tightens tolerance and synchronisation requirements, which can amplify jet-to-jet variability and complicate scale-up [202]. Commercial systems partly manage this by fixing dose and use conditions, but fixed-dose architecture inherently limits per-actuation volume and constrains indications.

### 5.2 Powder injectors

As needle-free powder injection (NFPI) technologies evolve, they offer a promising pathway to a safer and easier method for drug delivery. With future refinement, NFPI can serve a transformative role in the next generation of therapeutic and prophylactic measures. Despite growing interest in wearable drug delivery, no real-time wearable devices currently incorporate powder jet injection. Although this technique is promising for needle-free delivery, it remains largely confined to stationary or handheld systems. To explore its potential in miniaturised formats, attention has turned to portable devices such as gene guns, which have demonstrated success in both clinical and preclinical applications [46]. Such a notable example is the biolistic intradermal injector developed by Brouillette et al., which uses a miniature shock tube to propel microparticles at high velocities (Fig. 8D) [204]. The system employs high-pressure nitrogen gas (∼32 bar) released into a 19 mm driver section connected to a 6 mm × 51 mm test chamber. A double membrane separates these sections, filled with atmospheric air. While triggering, gas at ∼10.8 bar ruptures the membrane, generating unsteady flow and accelerating the particles through a geometric constriction between the sections. However, the reliance on external high-pressure nitrogen gas limits portability and poses safety and logistical challenges for clinical use.

Further advances in the aerodynamic design have also contributed to the development of more efficient NFPI systems. Liu et al. investigated the performance of transdermal biolistic drug delivery systems using supersonic nozzles, comparing two prototype devices: one using a converging–diverging supersonic nozzle (CDSN), based on the Venturi principle, and the other based on a contoured shock tube (CST) [205]. Fig. 8E shows the schematic of a hand-held system. Using CFD, the researchers characterised transient gas–particle dynamics and validated the simulations against experimental data such as pressure profiles and particle velocities. The findings showed that the CST design delivered particles more uniformly and with better control, achieving a mean velocity of approximately 699 m/s with minimal deviation. In contrast, the CDSN system exhibited greater velocity variation and flow disturbances due to shock-induced separation.

Soliman et al. reported another study involving CDSN. The team developed a novel power injection device for delivering DNA vaccines into human skin. Their system includes a C–D nozzle followed by a constant-area mixing duct, which accelerates helium gas to supersonic speeds. Powdered particles are introduced through a parallel inlet, which energises the boundary layer and helps to prevent flow separation. CFD simulations and semi-empirical calculations showed that gold (1.8 μm, 5 μm) and polystyrene (38 μm) microparticles could achieve penetration depths of 20–60 μm, sufficient for intradermal delivery [46]. As shown in Fig. 8F, particle velocity increased with higher driver pressures, but the system achieved effective delivery even at moderate values. Notably, the design achieved similar impact conditions at 33% lower gas pressure compared to previous devices. However, the reliance on helium as the driver gas poses a genuine limitation, as helium is costly, requires specialised storage, and is not widely accessible in clinical settings.

A novel advancement in biolistic drug delivery was introduced by Wijesundara et al., who developed a carrier gas–triggered biolistic system for transdermal delivery of DNA and protein therapeutics, in which biomacromolecules are encapsulated within zeolitic imidazolate framework-8 (ZIF-8) particles and delivered using a custom-built device termed the MOF-Jet [206]. The system employs a gas-driven mechanism to propel solid ZIF-8 particles into tissue, with release kinetics modulated by the carrier gas. A visual illustration of biomimetic mineralisation with ZIF-8 and biolistic delivery using the MOF-Jet is shown in Fig. 8G. Carbon dioxide creates a transiently acidic environment by interacting with tissue moisture, allowing for rapid particle dissolution and payload release, whereas compressed air maintains a neutral pH, enabling slower, sustained release. These gas-dependent release kinetics were confirmed in vivo, with the radiant efficiency of OVA-Cy7@ZIF particles declining more rapidly under CO_2_ than under compressed air, indicating faster protein release and clearance. This tunable release profile is a distinct innovation over traditional NFPI systems. Nonetheless, particle size variability and reliance on localised tissue conditions (i.e., hydration, pH) could bring in variability in delivery, and the CO_2_-elicited transient acidification could cause tissue irritation.

Solid-state formulations bring distinct constraints to NFPI. Powder properties such as polymorphism, hygroscopicity, and particle-size distribution can change during manufacturing and storage, and these shifts affect reconstitution behaviour, stability, and bioavailability of the device [210,211]. NFPI cartridges are also exposed to some additional risks, such as aggregation, electrostatic charging, and degradation under pressurised or low-humidity conditions, which can further compromise dose reproducibility. At the same time, delivery performance is highly dependent on gas dynamics and nozzle geometry, so small changes in these parameters or device handling can cause changes in penetration depth and dispersion. Although high particle velocities can be achieved, many platforms rely on high-pressure or specialised gases (e.g., nitrogen or helium), which reduces portability and clinical logistics [212]. Together, these factors explain why translation depends less on demonstrating penetration in proof-of-concept studies and more on establishing robust quality controls for both the powder and the device alongside scalability.

### 5.3 Iontophoresis-assisted drug delivery systems

The recent developments of iontophoretic technologies continued to expand from the typical current modulation and formulation approaches to directions of innovation in wearable integration and advanced material engineering. Such innovations are guiding iontophoresis into a bright future of becoming a smart, adaptive, and potentially even autonomous platform for transdermal drug delivery. One notable direction is using engineered drug matrices to improve delivery efficiency. For example, Morarad et al. developed a porous thermoplastic polyurethane (TPU) matrix for transdermal insulin administration, a notable step toward material-driven optimisation of iontophoretic delivery [213]. Using Triton X-100 as a porogen enabled tunable porosity, which directly enhanced electro-repulsive transport under an applied field. Drug release was assessed in Franz diffusion cells across pig skin under applied voltages (0–6 V), confirming that electric fields significantly enhanced insulin permeation compared to passive diffusion. Even with these encouraging results, concerns remain. Non-homogeneous porous structures may result from the particulate-leaching process, which could decrease the reproducibility of drug flux. Increasing porosity may weaken mechanical integrity and compromise long-term skin adhesion. These limitations could be addressed through precision pore engineering via phase-separation or templating techniques, alongside incorporating mechanically reinforcing yet conductive fillers (PRDOT: PSS fillers) to sustain integrity without sacrificing conductivity [214].

**Fig. 9:**
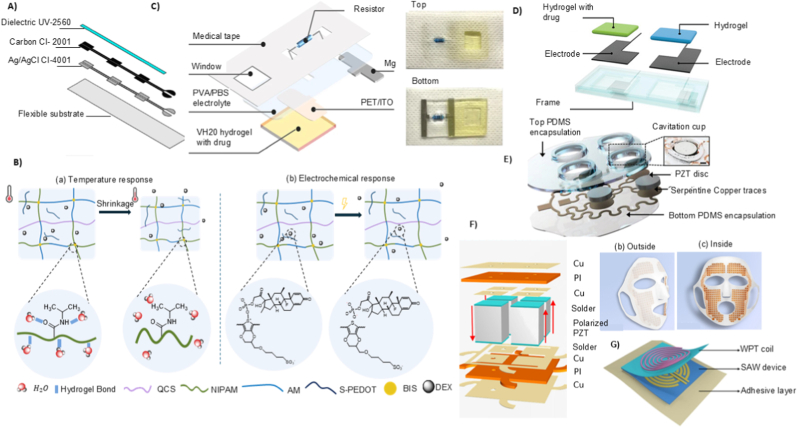
Performance characteristics of iontophoresis and sonophoresis based TNFDD systems: A) Illustration of a three-layer stack Electrode-printed on a flexible PET substrate, Reprinted with permission from Ref. [215]; B) Schematic diagram of drug release triggered by (a) thermal stimulation and (b) electrical stimulation [216]; C) Schematic circuit diagram and photographic images of the wearable iontophoresis patch powered by a magnesium (Mg) battery. [104]; D) Schematic illustration of the hydrogel based soft patch, Reprinted with permission from Ref. [217]; E) Exploded view of the 2D-array of cUSP (5 cm × 5cm × 2 mm) showing each constituent layer [218].; F) Visual representation of the SEFM: (a) inset showing a single island in the stretchable island–bridge mesh circuit, and (b, c) external and internal views of the SEFM. Reprinted with permission from Ref. [219]; G) Schematic illustration of SAW patch [220].

Beyond material-focused approaches such as porous TPU matrices, other efforts have concentrated on developing integrated, patient-ready platforms. Chaudon et al. reported a compact iontophoretic patch for treating and monitoring pressure ulcers [215]. This system utilised easily replaceable flexible electrodes and impedance sensing to monitor treatment progress. Using tolazoline as the therapeutic agent, the system achieved an eightfold increase in drug penetration compared to passive diffusion at a current of 250 μA. The electrodes comprised a three-layer stack (Fig. 9A) screen-printed on heat-stabilised PET (75 μm), with an Ag/AgCl layer (≈20 μm, 45:55 ratio) for ionic conduction, a carbon overlay (<20 Ω/sq) for pH protection, and a UV-cured dielectric coating for electrical insulation. This design offers biocompatibility as well as flexibility for the patch. However, the 30-min delivery time illustrates the need for further optimisation in terms of delivery speed and dosing precision. Similarly, Liang et al. developed a wearable iontophoretic patch for the treatment of atopic dermatitis [216]. The platform integrates an electroactive PEDOT-based polyelectrolyte hydrogel that responds to both body temperature and electrical stimulation. As demonstrated in Fig. 9B, the hydrogel network is contracted by thermal shrinking for drug release, while electrical stimulation drives electrostatic repulsion for further controlled delivery. By using a wearable zinc battery, the patch achieved a delivery efficacy of 68.4% under physiological temperature. However, the long-term stability of the hydrogel upon repeated thermal and electrical cycling has not been evaluated, which may cause structural fatigue, dehydration, or delamination and compromise the reliability of drug delivery. Notably, as reported by Gotovtsev et al., the dual-responsive PEDOT: PSS hydrogel relies on a porous, swelling network formed via freeze/thaw cycles, which, although effective for stimuli-responsive release, may be particularly susceptible to mechanical fatigue during repeated activation [221].

Zhou et al. introduced a simplified wearable iontophoretic patch with a built-in Mg battery (Fig. 9C) [104]. The use of a viologen-based hydrogel as both drug reservoir and cathode is particularly innovative, offering improved interface conductivity and energy density. However, integrating a discrete Mg battery directly into the patch may constrain mechanical flexibility compared to fully polymer-based platforms. A more futuristic approach is the development of self-powered iontophoretic systems. Wu et al. introduced a triboelectric nanogenerator (TENG)-based patch that harvests biomechanical energy from body motion to power transdermal drug delivery [217]. This eliminates the need for conventional or integrated batteries, addressing some of the bulk and rigidity concerns seen in Mg battery-powered systems. Integrated with a hydrogel-based electrode patch, this approach points toward fully autonomous wearable therapies. The schematic of the device structure and photographs of the fabricated device are shown in Fig. 9D. However, its reliance on motion input may limit its applicability for patients with restricted mobility. Implementing hybrid power approaches, where the biomechanical harvesting is supported by miniaturised and flexible storage elements for consistent performance across a wide range of patients.

Iontophoretic flux is often limited by competition from endogenous ions (Na^+^, K^+^, Cl^−^), which carry a large share of the applied current and reduce electromigration efficiency. At clinically tolerated current densities (≈0.3–0.5 mA cm^−2^), the effective transport number available to a cationic drug is frequently capped at ∼0.6–0.8 in physiological ionic environments, making a substantial fraction of current is inevitably “spent” on background ions unless the formulation suppresses co-ions. One possible way is to use salt bridges or compartmentalised donors to reduce electrode-derived ions, but this adds hardware complexity and can introduce long-term stability issues in wearable formats [50]. Electro-osmosis adds another layer of sensitivity: because its direction changes near the skin's isoelectric point (∼pH 4–4.5), modest pH shifts can either help or hinder delivery, sometimes independently of electromigration. In practice, many “wearable iontophoresis” demonstrations also incorporate microneedles, which improve flux but undermine the non-invasive premise by intentionally breaching the barrier [[222], [223], [224]]. Power is a second bottleneck; tethered supplies reduce wearability, although recent self-contained Mg-battery patches (areal energy ∼3.57 mWh cm^−2^) suggest that microampere-scale stimulation can be sustained for hours in thin, integrated devices.

### 5.4 Sonophoresis-assisted drug delivery systems

Miniaturisation has been one of the key challenges associated with wearable sonophoretic drug delivery devices. Conventional ultrasound devices are not portable because they have inbuilt transducers, drug reservoirs, and power supplies, making it impossible to use them for wearable applications. Recent developments have addressed these drawbacks and led to more flexible, wearable, and efficient ultrasound-mediated delivery systems. One of these notable advancements is the conformable ultrasound patch (cUSP) from Yu et al. for dermatological and cosmeceutical applications (Fig. 9E) [218]. The device incorporates piezoelectric transducers into a soft elastomer matrix, creating intermediate-frequency cavitation pockets (0.8 cm^2^, 1 mm deep) within a treatment zone of approximately 20 cm^2^. It enhances the transdermal transport of niacinamide by 26.2-fold in porcine skin models following a 10-min sonophoresis.

Although promising, cUSP operates at intermediate frequencies, which may restrict its ability to deliver larger hydrophilic molecules that benefit more from low-frequency cavitation. A promising direction would be to integrate frequency-tunable or hybrid acoustic modes, allowing cUSP-like platforms to strike a balance between effective cavitation and tissue safety. On the other hand, Zhai et al. introduced a hybrid method combining LFS with sponge Haliclona sp. spicules (SHS), referred to as cSoSp. The SHS produce nanochannels on the SC, whereas LFS creates cavitation-mediated lipid disruption and leads to synergistic increases in transdermal transport of fluorescent dextran (FD-4K) and low-molecular-weight heparin (LMWH) in animal models, without detectable skin irritation [225]. Although the dual-component system can achieve fine control of mechanical and acoustic effects for ideal penetration, it also presents difficulties for reproducible fabrication and alignment, potentially impairing uniformity of nanochannel generation and cavitation throughout the treatment site.

Another notable innovation is the Stretchable Electronic Facial Mask (SEFM), a skin-conforming device integrating sensors and ultrasound-assisted sonophoresis for facial drug delivery, developed by Li et al. [219] The device employs a dual-planar silicone design and a single-side soft pressing (SSSP) encapsulation technique, ensuring close contact with the skin while maintaining mechanical flexibility. Fig. 9F shows the external and internal views of the SEFM, highlighting the compact structural layout and arrangement of internal components, and provides a detailed view of a single island in the stretchable island-bridge mesh circuit, illustrating the conductor layout that enables mechanical flexibility without compromising electrical connectivity. However, the current mesh design can generate localised stress concentrations at the bridge junctions under repeated deformation. The minimal external encapsulation also leaves internal circuits vulnerable to environmental factors such as moisture or particulate contamination. To address these challenges, the bridge geometry could be optimised using serpentine or meander patterns to redistribute mechanical strain. A thin conformal polymer coating (e.g., parylene or PDMS) could be applied to improve environmental protection while preserving the flexibility [226,227].

Recent work shows the ability of surface acoustic waves (SAW) to enhance transdermal drug delivery. Zhang et al. reported a thin-film, wireless-powered SAW device to transport macromolecules such as hyaluronate rhodamine (5 kDa) transdermally, achieving ∼77.9% of the efficiency of cable-connected platforms [220]. Fig. 9G illustrates the schematic of the patch. Mechanistically, this system combines interfacial acoustic stimulation and micro-cavitation to enhance SC permeability. A critical engineering concern is the suboptimal coil coupling, where the efficiency drops sharply outside the 1.5 cm optimal distance, creating challenges for wearable or flexible configurations. It could be addressed by integrating a resonant multi-coil array or dynamic impedance-matching circuitry that actively tunes coupling in real time, maintaining near-maximal energy delivery regardless of coil misalignment or movement [228].

Ultrasound has reported an increase in transdermal transport, but the cavitation mechanisms that open pathways can also injure tissue if acoustic parameters are not properly controlled. LFS can produce very large enhancement factors (∼3 × to ∼10^3^ × over passive delivery), especially for hydrophilic or larger particles, but it often does so through highly non-uniform LTRs 229,230]. Without field homogenisation, this translates into hot spots and under-dosed zones. SAW-based patches improve wearability and can deliver macromolecules in a size- and depth-dependent way (e.g., ∼25–100 μm in porcine skin), but wireless implementations still pay an efficiency penalty (e.g., ∼77.9% of a cabled system) and remain sensitive to coupling and alignment [231]. These results suggest that clinical translation will depend less on proving ultrasound can enhance delivery, and more on reproducible “acoustic dosing” (frequency, intensity, duty cycle, exposure time), better spatial uniformity, and hybrid designs that combine ultrasound with a second mechanism to stabilise dose and depth.

### 5.5 Electroporation-assisted drug delivery systems

**Fig. 10:**
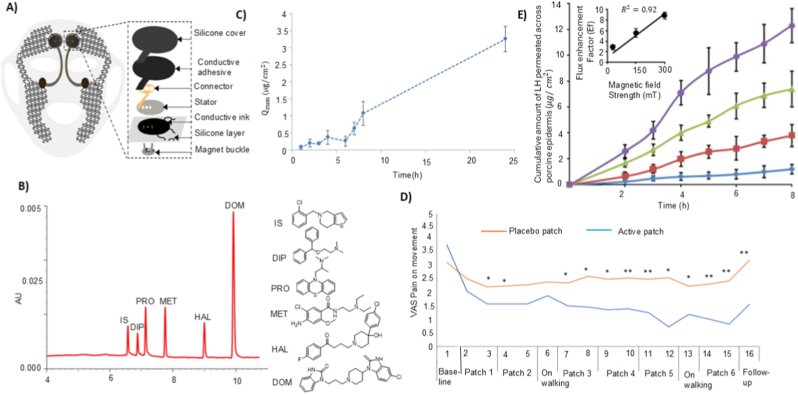
Performance characteristics of electroporation and magnetophoresis based systems in TNFDD: A) Disassembled structure of the Stretchable Electronic Facial Mask (SEFM), Reprinted with permission from Ref. [233]; B) Electropherogram of domperidone, haloperidol, metoclopramide, promethazine, diphenhydramine, and ticlopidine (internal standard obtained using capillary electrophoresis with hydrodynamic injection Reprinted with permission from Ref. [234]; C) Mean permeation profile of metoclopramide from Pentravan ® Plus, Reprinted with permission from Ref. [234]; D) Time-course of pain reduction, showing the difference in movement-related VAS ratings between placebo and active patches at all measurement points from baseline to 48-h follow-up. Reprinted with permission from Ref. [118]; E) Cumulative permeation of lidocaine across porcine epidermis under passive conditions and with magnetic field strengths of 30, 150, and 300 mT. Reprinted with permission from Ref. [79].

Developments in the electrode design, nanomaterials and integration with wearable systems have enhanced the therapeutic potential and translational relevance of electroporation. For instance, the hydrogel–electrode system of Kougkolos et al. integrates a nanocomposite hydrogel with conductive fillers, functioning as both a drug reservoir and an electroactive interface for electroporation [232]. It demonstrated non-uniform voltage distribution across skin layers, with low voltages permeabilising the SC and higher voltages (>300 V) reversibly disrupting viable epidermal cells. However, the reliance on elevated voltages would increase risks of Joule heating and electrochemical degradation. Moreover, the electrode's flat, planar design spreads current density inefficiently, thereby necessitating higher driving voltages. This could be addressed by engineering patterned electrode geometries (e.g., fractal or concentric ring layouts) to concentrate electric fields more effectively at the skin interface. Other than the hydrogel–electrode designs, efforts have shifted toward more wearable formats. The Stretchable Electronic Facial Mask (SEFM) represents one such approach, embedding conductive ink–modified electrodes into a silicone substrate to improve conformity and mechanical adaptability [233]. The device's disassembled structure is shown in Fig. 10A, highlighting the arrangement of electrodes and the flexible silicone mask that enables conformal skin contact and mechanical robustness. This wearable, reusable device demonstrated a 3–4-fold enhancement in niacinamide delivery in rats, with validation in human subjects.

The integration of electrophysical stimulation with advanced nanomaterials was explored by Simon et al., who developed a two-in-one electrode/reservoir composed of carbon nanotube-infused agarose hydrogel [235]. Their device enabled the delivery of macromolecules like 4 kDa dextran-FITC and revealed asymmetric permeation pathways through the stratum corneum. Notably, this research also indicated the superiority of electrophoresis for large molecules and for smaller molecules competing by diffusion, a differential that is crucial in picking the appropriate candidates for electro-mediated delivery. Building on the need for precise evaluation of drug transport, Mbaye et al. developed a capillary electrophoresis (CE) protocol optimised for quantifying antiemetic drug permeation across pig epidermis [234]. By integrating field-amplified sample injection with liquid–liquid extraction, the method enhanced detection sensitivity by 600- to 2000-fold, enabling accurate measurement of low-permeating drugs. Fig. 10B shows the electropherogram of domperidone, haloperidol, metoclopramide, promethazine, diphenhydramine, and ticlopidine (internal standard) obtained with hydrodynamic injection under the specified capillary and buffer conditions. Fig. 10C presents the mean permeation profile of metoclopramide from Pentravan®Plus across pig ear epidermis, highlighting the utility of this method for capturing drug transport kinetics. While not a delivery device per se, this high-sensitivity analytical platform is essential for assessing pharmacokinetics and optimising both device parameters and formulation design in electrophoretically assisted transdermal drug delivery systems.

Electroporation can deliver dramatic gains in skin transport, but that headline performance is also what makes it hard to translate. Once pulse amplitudes exceed the SC breakdown threshold (often discussed as ∼75–100 V across the SC), flux enhancements can reach ∼10^4^-fold for some compounds, yet small changes in skin impedance (between donors and even body sites) can shift the actual field delivered and therefore the dose 236. This creates a real dosimetry problem for wearables: repeated high-voltage pulse trains can cause irritation or discomfort, and complex hydrogel/electrode architectures add manufacturing and usability burden 237,238]. In practice, the main barrier is not “can it work?” but “can it be controlled and reproduced?”—which requires clearer reporting and control of electrode geometry, pulse waveform, and pulse train, ideally with impedance-guided feedback and modelling to keep thermal and electrochemical loads within safe limits [[239], [240], [241]].

### 5.6 Magnetophoresis-assisted drug delivery systems

Magnetophoresis is highly compatible with wearable devices and transdermal patches. Because the side effects of magnetic fields on the skin are nearly negligible, magnets can be placed near or attached to the skin without any safety concerns and can also eliminate the need for sterilisation [242]. Another advantage of using magnets in transdermal drug delivery is their versatility. They can be easily moulded into different shapes and sizes, and permanent magnets operate without external power or prior charging. Recent advancements in magnetophoresis have translated beyond in vitro and mechanistic studies into the development of wearable and clinical drug delivery systems. A phase I clinical study by Wright et al. introduced a novel magnetophoresis-enhanced transdermal delivery system for ibuprofen in patients with painful knee osteoarthritis (OA) [118]. Utilising a crossover design, the study compared the analgesic efficacy of magnetophoretic ibuprofen patches versus placebo. The wearable patch system employed magnetic technology to improve skin permeability and accelerate local drug absorption. The results demonstrated a statistically significant reduction in movement-related pain as assessed by the VAS and WOMAC scales. Fig. 10D shows the time history of this reduction is shown in, where the difference in pain on movement VAS ratings between placebo and active patches is presented across all measurement time points from baseline to 48-h follow-up. Even though the study showed analgesic effects, the absence of pharmacokinetic data limits understanding of systemic absorption and distribution. Another clinical investigation explored the combined application of pulsed low-frequency magnetic fields (PEMFs) and transdermal diclofenac in patients with knee osteoarthritis. In a randomised, placebo-controlled trial involving 65 patients, this wearable device delivered diclofenac transdermally under the pulsed magnetic field with an intensity of 20 mT and frequency of 6.25 Hz. It has been reported that there has been a significant reduction in pain and stiffness, as measured by VAS, WOMAC, and EQ-5D scores [243]. Notably, 67.8% of patients met the OMERACT-OARSI responder criteria, indicating clinically meaningful improvement. However, similar to the study reported by Wright et al., pharmacokinetic measurements were not collected, limiting technical understanding of systemic absorption and drug distribution.

Murthy et al. engineered a reservoir-type transdermal patch with an integrated magnetic backing for the delivery of lidocaine. In-vitro studies conducted across porcine skin demonstrated a clear correlation between magnetic field strength (30, 150, and 300 mT) and enhancement in drug flux [79]. This relationship between magnetic field intensity and flux enhancement is illustrated in Fig. 10E, which depicts the flux enhancement factor as a function of magnetic field strength. The experimental setup used a porcine epidermis sandwiched between a donor chamber containing the drug solution and a receiver chamber filled with isotonic saline, with two neodymium magnets positioned on either side of the donor compartment and Ag/AgCl electrode wires measuring electrical resistance. In vivo studies in rats further confirmed the superior dermal bioavailability of the magnetophoretic patch over non-magnetic controls [79]. This system presents a promising low-power solution for enhancing transdermal drug delivery. However, the use of a static magnetic field limits tunability and adaptability across different drug profiles.

Mechanistically, magnetophoresis still sits on less-settled ground than most energy-based TNFDDS, the weight of evidence suggests little direct SC damage, with effects more consistent with field-driven transport (e.g., diamagnetic/magnetohydrodynamic contributions) that may vary with drug chemistry and skin type. Even where flux increases are reported at ∼30–300 mT, performance is hard to generalise because the “dose” is not just field strength; it depends on field geometry (uniform vs gradient), exposure time, and patch architecture, so small design changes can shift outcomes and limit reproducibility [79]. Clinically, the current evidence base is still thin and often relies on symptom scores rather than paired PK/dermatokinetic measurements, which makes it difficult to separate true exposure gains from placebo or local effects and to build a dose–response argument [118]. For wearable translation, the trade-off is clear: permanent magnets are simple and power-free but fixed, whereas PEMF offers tunability at the cost of bulk and power management. Finally, fields in this range appear well tolerated in short-term use, but longer-term safety, especially in patients with implanted devices, needs more explicit evaluation [244].

### 5.7 Thermal ablation-assisted drug delivery systems

**Fig. 11:**
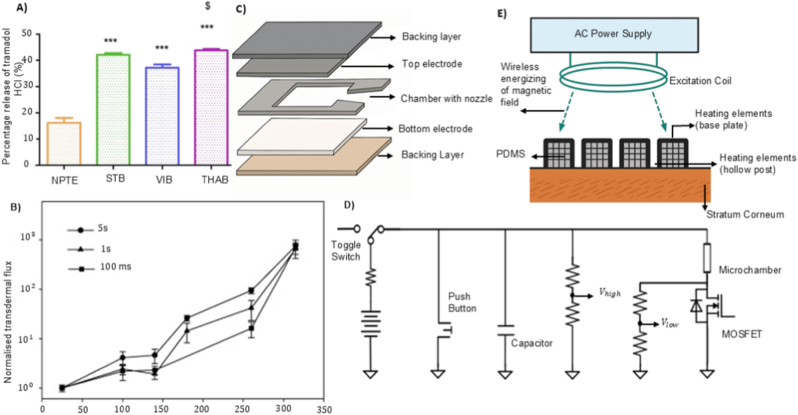
Performance Characteristics of Thermal ablation in TNFDD: A) Percentage release of tramadol HCl under different treatments (NPTE, STB, VIB, THAB). Reprinted with permission from. [245]*;* B) Effect of temperature and heating time on calcein flux across human epidermis. Reprinted with permission from Ref. [246].; C) Schematic illustration of the five-layer microchamber structure [86]; D) The electrical circuit used to generate an electrical discharge within the microchamber [86]; E) Schematic of a wireless inductive heating system for micro-ablation of the stratum corneum [247].

Rather than bypassing the barrier entirely, thermal ablation removes microdomains of the SC in a controlled manner, creating transient microchannels that facilitate molecular diffusion. Supporting this principle, Macit et al. investigated the effects of mild physical triggers, such as steam bath (STB), vibration (VIB), and thermal ablation (THAB) at 42 °C, on the systemic absorption of tramadol HCl in Wistar rats [245]. As illustrated in Fig. 11A, controlled thermal ablation (THAB, 42 °C) produced the highest enhancement in tramadol permeation, followed by steam bath (STB) and vibration (VIB), all of which were statistically significant compared to untreated controls. All three interventions significantly increased plasma drug levels compared to control, with THAB showing the greatest enhancement (43.8%), closely followed by STB (42.1%) and VIB (37.2%). These results underscore the sensitivity of the skin to external stimuli and validate the use of controlled thermal inputs to improve transdermal delivery. Among the most studied ablation platforms is the PassPort® system, which employs an array of micron-scale metallic filaments termed “porators.” Upon activation, a brief electrical pulse is delivered to the filaments, which generate heat through resistive (Joule) heating [93]. The generated thermal energy is transferred to specific skin contact points, ablating the stratum corneum to a controlled depth (∼50 μm). This penetration is deep enough to extend down to the viable epidermis but not through the dermis and does not induce any discomfort [248]. The microporation-created micropores are closed off by interstitial fluid and develop aqueous channels that admit hydrophilic molecules and transmigrate through the barrier to the systemic circulation through lymphatics and dermal capillaries. This method has also been particularly notable in the delivery of biologics and other poorly absorptive molecules through traditional patches.

In one study, Park et al. developed a device that delivered short, high-temperature pulses to skin by mounting skin samples on a solenoid-driven plunger that briefly contacted a constant-temperature heat source (hot plate for 30–250 °C; soldering iron for 176–450 °C) [246]. Exposure times were precisely controlled (100 ms to 5 s) using a digital timer, enabling reproducible delivery of thermal pulses without sustained heating of underlying tissue. Using this setup, human and porcine cadaveric skin was exposed to heat sources ranging from 100 to 315 °C. As demonstrated in Fig. 11B, skin permeability was governed more strongly by temperature than by duration of heating. However, the study does not report quantitative measures of intra- or inter-trial variability, nor does it detail how uniform contact between the plunger and skin surface was ensured. To overcome the limitations of contact-based systems, newer device architectures have been developed. Lee et al. developed a microsecond thermal ablation device that generates ultra-short (∼100 μs) bursts of superheated steam by electrically discharging a microchamber filled with water [86]. The chamber, fabricated via laser micromachining and lamination of polymer and metal layers, ejects steam through a tapered nozzle, with activation confirmed by voltage, current, and force measurements showing peak ejection around 100 μs. The schematic diagram of the microchamber, illustrating its five distinct layers and the electrical circuit used to generate the electrical discharge, is shown in Fig. 11C and D. Two mask designs are employed: a conductive mask for debris-free thermal transfer and a window mask (∼100 μm holes) for lateral control, enabling precise three-dimensional ablation of the stratum corneum while preserving deeper epidermal layers. Finite element modelling and temperature measurements suggest ejectate temperatures between 290 °C and 1035 °C, which are sufficient for selective barrier disruption without significant heating of the underlying tissue. The device achieved >1000-fold enhancement in permeability for both small (sulforhodamine B) and large (BSA, 66 kDa) hydrophilic molecules in human and porcine cadaver skin. Even though the study demonstrates precise temporal and spatial control, the reported upper range ejectate temperature assumes ideal energy conversion with no losses, which likely overestimates the actual temperature delivered to the skin. This raises concerns about reproducibility and device safety in vivo, as real-world thermal exposure may vary significantly across microchambers or batches.

In another innovative approach, Park et al. developed a wireless thermal micro-ablation system utilising nickel micro-heating elements on a stainless-steel substrate, which is heated via an alternating magnetic field (Fig. 11E) [247]. The Type B hollow post array, insulated with PDMS, localised heat to the tips, producing micron-scale pores in the stratum corneum, as confirmed by in vitro human skin studies and SEM imaging. Finite element simulations and liquid crystal paper tests demonstrated rapid heating, with higher frequencies increasing power density. The system is wireless, patch-compatible, and offers potential for remotely controlled, wearable transdermal drug delivery. Non-uniform heating across the array may cause variability in pore sizes, and in vivo safety and reproducibility remain to be established. However, finite element analysis revealed non-uniform eddy current distribution within the hollow post geometry, leading to edge-dominated heating and potentially heterogeneous ablation profiles. Furthermore, the reported experiments relied on temperature-indicating papers rather than direct thermometric measurements, leaving uncertainty regarding precise thermal dose delivery at the skin interface.

Thermal ablation can increase skin permeability dramatically, but it only works clinically if the thermal dose is tightly controlled. The aim is simple: create micropores in the SC while avoiding heat spread into deeper tissue. Microsecond systems show the potential, when heating is limited to ∼100 μs, permeability increases of >10^3^-fold have been reported for both small dyes and proteins (e.g., BSA, 66 kDa) in cadaver skin [86]. The challenge is repeatability because heat transfer changes with skin thickness, hydration, contact pressure, and small non-uniformities across heater arrays, so pore size and depth can drift between users and even between applications. Wearable use makes this harder because motion and changing contact can alter the delivered dose. For reliable performance, devices need feedback at the skin interface (e.g., temperature or impedance), and studies should consistently report the key dose descriptors, peak surface temperature, pulse width, spot size, and contact conditions, so results can be compared and reproduced.

### 5.8 Laser ablation-assisted drug delivery systems

**Fig. 12:**
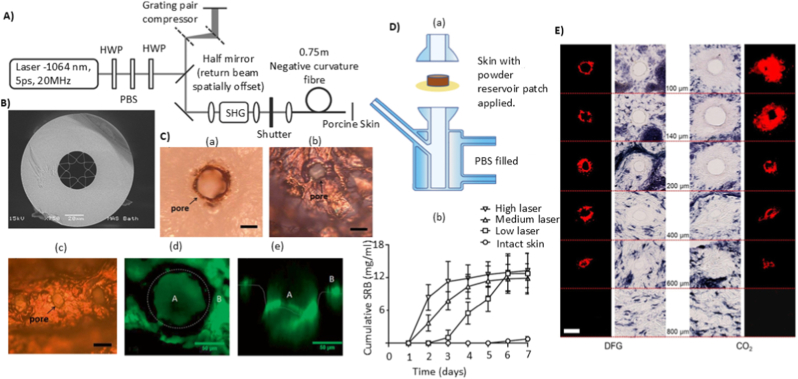
Performance characteristics of laser ablation in TNFDD: A**)** Experimental setup with frequency doubling and pulse compression (HWP: half-wave plate, PBS: polarising beam splitter, SHG: second-harmonic generation); B) SEM image of a hollow-core negative curvature fibre (HC-NCF) used for beam delivery; C) Optical microscopy of pores formed in uninked and inked skin by direct focusing or HC-NCF delivery. Reprinted with permission from Ref. [249]; D) (a) Schematic of the Franz cell setup for AFL-assisted powder reservoir patch delivery on treated or intact skin. (b) Cumulative SRB delivery in recipient chambers was measured daily for 7 days (n = 6). Reprinted with permission from. [250]; E) Horizontal sections of ex vivo human abdominal skin after treatment with DFG and CO_2_ lasers (100–800 μm depth). Stained images. Reprinted with permission from Ref. [251].

An emerging strategy in laser ablation–mediated transdermal drug delivery involves the use of femtosecond pulsed fibre lasers delivered via hollow-core negative curvature fibres (HC-NCFs). Garvie-Cook et al. demonstrated that this configuration enables highly controlled skin microporation with minimal collateral thermal damage, owing to the ultra-short pulse durations that confine energy deposition to targeted regions [249]. The experimental setup incorporated frequency doubling and pulse compression **(**Fig. 12A), with beam delivery via a hollow-core negative curvature fibre (SEM in Fig. 12B). Remarkably, pre-application of an ink layer enhanced the efficacy of poration by facilitating plasma formation through thermionic electron emission, thereby reducing the laser energy threshold required (Fig. 12C). Raman spectroscopic analysis confirmed the absence of significant thermal diffusion into surrounding tissues. Moreover, in vitro permeation studies revealed substantially high transdermal delivery of hydrophilic molecules under these conditions. A key limitation lies in the dependency on external ink to initiate plasma, which could introduce variability in pore formation and difficulty in being clinically practical. Furthermore, femtosecond systems remain costly and complex, restricting their translational potential. A promising solution would be to replace exogenous ink with biocompatible chromophores or nanoparticle sensitisers that selectively localise within the stratum corneum, thereby lowering the energy threshold [252]. Complementary to this precision approach, Kakar et al. introduced an innovative approach that combined ablative fractional laser (AFL) pretreatment with powder reservoir patches for sustained transdermal delivery [250]. AFL-generated microchannels enabled the gradual dissolution of drug-coated powders by trans epidermal water loss, allowing week-long release of hydrophilic compounds such as zidovudine and bovine serum albumin, with patch loading up to 70 mg per 0.5 cm^2^. In vivo studies demonstrated dual-phase pharmacokinetics and systemic delivery lasting up to six days, while safety assessments confirmed rapid microchannel resealing and complete skin recovery within three days after patch removal (Fig. 12D). A major advantage of this platform is its simplicity; drug powders can be directly coated without complex formulations. However, the dependence on high dose loading and variability in water evaporation rates may pose translational challenges. Future optimisation could focus on controlled-reservoir patch designs or humidity-modulating backings to standardise dissolution dynamics and extend clinical applicability. In this context, T. Suwan et al. conducted a comparative ex vivo study of a clinical CO_2_ laser (10 600 nm) and a prototype 3050/3200 nm fibre-pumped difference frequency generation (DFG) laser for laser-assisted drug delivery [251]. Both systems generated microchannels that enabled topical nanoparticle and dye penetration, but the DFG laser produced more uniform channels with a smaller spot size (∼40 μm) and markedly thinner coagulation zones (Fig. 12E). At equivalent treatment densities, the DFG system achieved significantly higher total fluorescence intensity and more consistent uptake across skin depths, indicating improved delivery efficiency. These features suggest that DFG lasers may enhance drug diffusion while reducing tissue damage and recovery time compared to CO_2_ systems. Overall, DFG lasers appear to improve the “delivery–damage” balance. At matched depths, they produced smaller channels (∼56 μm) and thinner CZ (∼22 μm) than CO_2_ (∼76 μm; ∼67 μm CZ) while increasing uptake of a 30 kDa tracer [251]. Benchmarking trends point the same way, so spot size and CZ thickness should be treated as core comparators, not optional details. AFL plus powder-reservoir patches can push delivery to ∼1 week at high loading (∼35–70 mg/0.5 cm^2^), but future studies need PK plus standardised laser-dose reporting to support clinical translation [250].

### 5.9 Radio frequency ablation-assisted drug delivery systems

Unlike iontophoresis or electroporation, radio frequency (RF) ablation enables the delivery of a broad range of compounds, irrespective of charge. Moreover, the microchannels generated by the ablation are not as sensitive as nociceptors and are superficial, rendering this procedure significantly less painful. Clinically, RF ablation has proven efficacy in boosting the percutaneous absorption of peptides, vitamins, and hyaluronic acid, and has been accompanied by positive dermatological impacts, including collagen remodelling and skin rejuvenation [253]. For instance, in cancer studies, RF ablation has been used to locally trigger drug release from thermosensitive liposomes in liver tumours, demonstrating enhanced intratumoral accumulation and therapeutic efficacy [254]. Although such approaches do not involve the skin barrier, they exemplify the versatility of RF energy as a spatially controlled drug delivery trigger. Several RF ablation devices have been developed over the past two decades to enable controlled transdermal delivery of diverse therapeutic agents. The underlying principle across all these systems is the creation of transient micropores in the SC using thermally mediated RF energy, which enhances skin permeability without compromising deeper dermal layers. One of the earliest technical demonstrations of this was by Levin et al., who used an RF device to create a uniform array of microchannels in rodent skin, enabling efficient transdermal delivery of human growth hormone (hGH) [94]. Notably, this study established a fundamental technical benchmark: RF-generated microchannels of uniform diameter and spacing could support systemic absorption of macromolecules (22 kDa) with high bioavailability (up to 75%) and preserved bioactivity.

Building on this concept of controlled pore formation, Lee et al. advanced RF microporation by introducing a bipolar fractional RF device that delivered energy through a 64-microelectrode array, creating uniform micropores (∼170 μm diameter, ∼35 μm depth) in a pixelated pattern [96]. This configuration limited coverage to <5% of the skin surface per pass, thereby preserving barrier integrity and supporting rapid recovery. The system significantly enhanced macromolecular transport, with peptide delivery increased up to 23-fold and detectable penetration of siRNA (∼10–15 kDa) and dextrans as large as 40 kDa. However, pore depth was relatively shallow, and residual stratum corneum occasionally reduced uptake, while erythema indicated localised heating effects. Optimising electrode design and integrating real-time impedance feedback have been suggested as ways to improve pore uniformity and minimise variability. Kim et al. extended fractional RF microporation to both macromolecules (FITC-dextrans up to 40 kDa) and cosmeceutical actives such as α-bisabolol, arbutin, and EGF, demonstrating its versatility beyond systemic delivery [255]. Their 100-electrode array reproducibly generated microchannels (∼70–100 μm deep), with transport strongly influenced by molecular size, polarity, and pore hydrophilicity. In vitro, RF pretreatment enhanced delivery up to 6.8-fold, and in vivo, it reduced UVB-induced pigmentation and wrinkle formation, with EGF-treated skin showing improved collagen remodelling and barrier repair. However, the shallow pore depth limited the delivery of very large biomolecules, and localised erythema indicated residual thermal stress. This could be eliminated by including real-time impedance-guided feedback to fine-tune ablation and engineered electrode geometries for more uniform pores.

Overall, fractional RF microporation appears to have a practical operating window: it can reproducibly open superficial microchannels that support delivery of peptides and mid-sized macromolecules (around ∼20–40 kDa), with high bioavailability reported in selected models (up to ∼75%) [256]. But the limits are just as important—delivery drops with increasing size, shallow pores and residual stratum corneum can cap uptake, and erythema signals that thermal stress is still a real constraint. To make this clinically convincing, “fold-increase” data should be backed by dose-relevant endpoints and reproducibility: standardised reporting of pore geometry/coverage (ideally with dermoscopy or OCT), barrier recovery (e.g., TEWL), and matched dermatokinetic/PK readouts (tape stripping, microdialysis, and/or plasma PK). At the device level, electrode geometry and impedance-guided control should be treated as critical quality attributes, because they govern not only efficacy but consistency and safety across variable skin and real-world use.

## 6 -Translational barriers and regulatory considerations for TNFDDS

TNFDDS rarely reach approval, not because delivery is impossible, but because regulators require proof of consistent performance under real-world variability [34,257]. Unlike a hypodermic needle, which provides a direct route into a defined compartment, TNFDDS rely on an energy-mediated event to bypass the SC and place the drug at an intended depth and spread. Dose consistency is a major challenge because skin thickness, hydration, elasticity, and subcutaneous structure vary across people and body sites, and scarring or hair follicles can further alter penetration and drug spread. At the same time, minor changes in actuator output or device–skin contact conditions, including applied force and angle, can alter the delivered depth and distribution under-delivery risks, loss of efficacy, whereas excessive penetration increases the likelihood of pain, bruising, bleeding, or unintended deep delivery. TNFDDS also introduce failure modes that are tightly linked to performance, such as incomplete actuation, output instability, leakage, and clogging, all of which can reduce delivered dose and repeatability. In addition, where self-administration is an intended use, usability becomes a technical determinant of both safety and performance because user errors can directly change device–skin coupling and therefore delivery outcomes [258]. Together, these barriers form the “Valley of Death” for TNFDDS. In the following subsections, these barriers are discussed through five bottlenecks: product definition and regulatory pathway selection, design and development controls, limitations of preclinical models and standardisation, manufacturing scale-up and scalability, and cost-effectiveness and commercial adoption.

### 6.1 Product definition and regulatory pathway

Many TNFDDS stall late in development because regulatory strategy is treated as paperwork instead of a design decision. If the product is not classified early as a drug, a device, or a combination product, developers may generate evidence that does not fit the final pathway and may need to repeat studies later. Since TNFDDS outcomes arise from the combined action of formulation and device, regulators place particular weight on the primary mode of action (PMOA) [259,260]. PMOA reasoning supports the assignment of a lead FDA centre for premarket review, and when PMOA is uncertain, FDA provides a formal route to obtain a binding determination through the Request for Designation (RFD) process [261]. These pathway choices have practical consequences for development. Drug-led programmes typically start clinical studies under an Investigational New Drug (IND) application, whereas device-led studies may proceed under an Investigational Device Exemption (IDE) [262,263]. For marketing, devices may follow the 510(k) route or require Premarket Approval (PMA), depending on risk and the strength of evidence needed [264,265].

### 6.2 Design and development controls

Once the pathway is clear, the next translational hurdle is demonstrating that performance and safety are controlled by design, not dependent on ideal laboratory conditions. A practical approach is to define measurable design inputs early through a target product profile (TPP). For TNFDDS, this typically includes target dose and allowable dose error, intended delivery depth or tissue compartment, dose volume and concentration range, dose time, acceptable pain and skin response limits, and the intended users and user setting (home or clinic). Risk management should be presented as the engineering logic that links hazards to technical causes and to specific controls (aligned with ISO 14971) [266]. For TNFDDS, usability engineering should be treated as part of this control strategy, because use errors can directly alter device–skin coupling and therefore dose delivery; IEC 62366-1 frames usability engineering as a safety-related process to analyse and mitigate risks arising from correct use and use errors under normal use [267]. Material choice must also be controlled because skin-contacting materials require biological evaluation within a risk management framework (ISO 10993-1), and drug-contacting materials can affect dose accuracy and stability through adsorption, swelling, or extractables/leachables [268].

### 6.3 Preclinical model limitations and standardisation challenges

Preclinical testing is essential for TNFDDS, yet the predictive value of commonly used skin models is constrained by interspecies differences, anatomical variability, and limited standardisation across laboratories. These constraints matter more for TNFDDS than for many passive systems because delivery outcomes are shaped not only by barrier properties of the SC, but also by tissue biomechanics and the device–skin interaction during actuation [269]. Human excised skin is the closest model we have for transdermal testing, and it can give results that track well with in vivo data. However, skin thickness, hydration, lipid composition, and stiffness can all vary across the anatomical site. Moreover, skin characteristics differ across donors, including factors such as age, genetics, overall health, and lifestyle [270]. If the skin specimen is poorly selected or insufficiently qualified, penetration depth and dispersion may deviate from expected profiles, increasing the chances of tissue injury. Reporting the skin source, body site, preparation (e.g., dermatomed to 500 μm), and storage conditions (fresh, frozen, hydration state) should be mandatory. If these factors are not controlled, two studies may differ not because the devices differ, but because the skin conditions do [271].

Animal skin is an alternative that is used in preclinical testing because of the limited accessibility to human skin. Porcine skin is commonly used as a structurally similar substitute. However, factors such as appendage density, permeability and stiffness can differ and influence penetration depth. Moreover, follicles and glands may vary from human skin, affecting transport pathways and local dispersion [272]. As a result, regulators generally view animal data as supportive rather than fully predictive of human performance. Ex vivo diffusion cells (most commonly Franz cells) are considered a valuable method for transdermal evaluation because they provide a controlled setting to compare formulations and devices. Zorec et al. conducted their transdermal electroporation study using vertical glass Franz diffusion cells with dermatomed porcine skin, demonstrating that this setup is suitable for quantifying molecular transport across skin under controlled in vitro conditions [273]. This general approach is formalised in OECD Test Guideline 428, which sets out principles for in vitro skin absorption using excised skin and emphasises methodological consistency [274]. However, diffusion data sets obtained from Franz diffusion cell experiments are generally interpreted as comparative indicators under controlled conditions rather than as direct predictors of in-vivo transdermal performance, because electroporation-mediated delivery involves localised microdisruption and depth-dependent deposition patterns that are strongly influenced by tissue mechanics and electrical behaviour at the device–skin interface. In spite of the limitations in preclinical testing, standardisation became necessary to make sure the data are reliable [275]. The value of preclinical transdermal data depends heavily on strict standardisation and rigorous membrane qualification. Current regulatory expectations, such as the FDA's IVPT guidance for topical products, specifically recommend excised human skin and each section undergo barrier-integrity verification through methods such as tritiated water permeation, trans epidermal water loss (TEWL), or electrical impedance or conductance testing [276]. These checks ensure that the skin barrier is intact and suitable for permeation studies.

### 6.4 Manufacturing challenges and scalability

For energy-driven delivery platforms, small changes in component tolerances, assembly alignment, sealing behaviour, and material ageing can translate into measurable shifts in output characteristics. In practice, manufacturability must be treated as a performance requirement: the device is only clinically credible if the same delivery behaviour can be produced consistently across lots and maintained throughout the product lifetime. A second scalability barrier is that TNFDDS often sit at a drug–device interface, where both formulation attributes and device attributes contribute to clinical performance. Changes in the formulation such as viscosity, concentration, particle content, or stability, can reduce the dose consistency. Similarly, changes in polymers, lubricants, reservoirs, or fluid-path materials can cause inaccuracies in dosing because the drug may stick to surfaces, cause swelling, increase friction, or even leak. As a result, scale-up requires a disciplined change-control strategy because minor material substitutions can meaningfully affect performance equivalence. The sterilisation method has to keep the product sterile without damaging its mechanical or functional performance, and the packaging must remain intact through shipping, handling, and the entire shelf life [277,278].

### 6.5 Cost-effectiveness and commercialisation constraints

Even when the regulatory pathway is clear and technical performance is convincing, adoption of TNFDDS depends on whether the technology delivers value beyond standard needle–syringe administration. In most healthcare settings, the comparator of TNFDDS is inexpensive and familiar, which makes it difficult to justify switching unless TNFDDS bring clear extra benefits [279]. For example, Australian pharmacy vaccination programmes reimburse an administration fee of A$20.05 per dose (from July 1, 2025), which far exceeds the cost of the needle–syringe consumable [280]. A recent economic analysis of fractional-dose IPV delivery using the marketed PharmaJet Tropis platform reported an injector cost in the range of US$291–415 and single-use consumables priced at approximately US$0.34–0.59 per syringe, plus an adapter cost of roughly US$0.38–0.45 [281]. Despite higher device-related consumable costs than needles and syringes, the study estimated net savings of about US$0.07–1.00 per administered dose when Tropis enabled fractional dosing, depending on vaccine wastage and operational assumptions. In this way, TNFDDS are most likely to be cost-effective when they unlock system-level benefits that are large enough to offset device cost-of-goods and implementation burden, such as dose-sparing, improved coverage or throughput, reduced sharps and needlestick burden, or improved adherence in self-administration settings.

## 7 Conclusion and outlook

Transdermal needle-free drug delivery technologies stand out for their potential to deliver a wide range of therapeutics, including biologics, vaccines, and small molecules, without the pain, infection or trauma associated with needles. The evolution of TNFDDS is associated with the advances in engineering, changes in patient expectations, and the rise of decentralised and personalised healthcare. However, despite notable progress, many current devices remain bulky and mechanically complex, limiting their appeal for long-term use. Moreover, the dosage efficiency remains relatively low in most reported needle-free drug delivery devices, and instead of actively enhancing skin permeation, many of them still rely on passive diffusion for drug delivery.

Only a few devices utilise standalone skin permeation techniques, and some are described as “needle-free” only after integration with microneedles. We emphasise that neither microneedles nor many so-called needle-free enhancement methods are strictly non-invasive: both intentionally perturb the stratum corneum to enable transport. Importantly, the degree of barrier disruption and recovery kinetics vary substantially across modalities, ablative approaches can cause more persistent barrier impairment than microneedle-formed microchannels, whereas electrical- or acoustic-based methods may achieve enhancement with comparatively limited structural disruption depending on operating conditions. Accordingly, device classification should reflect the underlying mechanism and expected barrier recovery rather than the presence/absence of a hypodermic needle. Even though numerous TNFDD systems have been reported in this paper, only a few are integrated with biosensing capabilities. The next generation of wearable drug delivery devices should be capable of monitoring biological parameters and delivering therapeutics in real time, a feature that is still lacking in current research. The future of TNFDDS lies not only in improving delivery efficiency but in rethinking device design to prioritise comfort and seamless integration with daily life. This review has underscored the different penetration methods and their critical role of activation mechanisms, such as jet injection, iontophoresis, and ultrasound, in shaping delivery performance and patient experience. The convergence of bioengineering, materials science, and microelectronics has enabled the development of programmable, wearable systems that respond to individual needs. Yet, challenges persist, including formulation stability and miniaturisation of power sources. From a formulation and CMC perspective, this review clarifies how each modality's operating conditions constrain formulation design, particularly dose reproducibility, local tolerability, and storage robustness. Recent developments in flexible and stretchable microfluidics, especially the emerging field of micro elastofluidics, offer solutions to these challenges. These systems work on the interaction between fluid flow and elastic materials at microscale, enabling compact and conformal devices that have better interface with biological tissues. Innovations such as elastic capsules for drug storage, or viscoelastic fluids to improve fluid mixing and particle separation, pave the way for fully integrated, skin-mounted drug delivery platforms that can also monitor physiological signals in real time. Moreover, combining such systems with biosensors, and feedback control could enable responsive, closed-loop drug delivery. The use of artificial intelligence and machine learning can further personalise treatment by adapting dosing to real-time biomarker data, while cloud integration through IoT/IoMT infrastructures supports remote therapy and precision medicine.

## Fundings

The project is supported by the 10.13039/501100000923Australian Research Council through the Australian 10.13039/100019505Laureate Fellowship (FL230100023) to N.T.N.

## CRediT authorship contribution statement

**Apoorva Sasikala:** Data curation, Formal analysis, Investigation, Methodology, Visualization, Writing – original draft. **Du Tuan Tran:** Methodology, Writing – review & editing. **Jun Zhang:** Supervision, Writing – review & editing. **Nam-Trung Nguyen:** Conceptualization, Funding acquisition, Investigation, Project administration, Resources, Supervision, Writing – review & editing.

## Declaration of competing interest

The authors declare the following financial interests/personal relationships which may be considered as potential competing interests: Nam-Trung Nguyen reports financial support was provided by Australian Research Council. If there are other authors, they dlare that they have no known competing financial interests or personal relationships that could have appeared to influence the work reported in this paper.

## Data Availability

No data was used for the research described in the article.

## Abbreviations

AC: Alternating Current
AP: Alternating Polarity
CDSN: converging–diverging supersonic nozzle
CFD: Computational Fluid Dynamics
CST: contoured shock tube
cUSP: Conformable Ultrasound Patch
NFPI: Needle-Free Powder Injector
NIR: Near Infrared
PCL: Polycaprolactone
PDL: Poly-dl-lactic acid
PDMS: Polydimethylsiloxane
PEG–PLGA: Poly (ethylene glycol)-block-poly (lactic-co-glycolic acid)
PEMFs: Pulsed Electromagnetic Fields
PZT: : Lead Zirconate Titanate
RF: : radiofrequency
SAW: : surface acoustic waves
SC: : Stratum Corneum
SEFM: Stretchable Electronic Facial Mask
TDD: : Transdermal Drug Delivery
TNFDDS: Transdermal Needle-Free Drug Delivery Systems
TPU: : Thermoplastic Polyurethane
VAS: : Visual Analogue Scale
WOMAC: Western Ontario and McMaster Universities Osteoarthritis Index
WPT: : Wireless Power Transfer
